# Sepsis Stewardship: The Puzzle of Antibiotic Therapy in the Context of Individualization of Decision Making

**DOI:** 10.3390/jpm14010106

**Published:** 2024-01-18

**Authors:** Fernando Ramasco, Rosa Méndez, Alejandro Suarez de la Rica, Rafael González de Castro, Emilio Maseda

**Affiliations:** 1 Department of Anaesthesiology and Surgical Intensive Care, Hospital Universitario de La Princesa, Diego de León 62, 28006 Madrid, Spain; rmendezh@salud.madrid.org (R.M.); alejandro.suarez@salud.madrid.org (A.S.d.l.R.); 2 Department of Anaesthesiology and Surgical Intensive Care, Hospital Universitario de León, 24071 León, Spain; rgonzalezdc@saludcastillayleon.es; 3 Department of Anaesthesiology and Surgical Intensive Care, Hospital Universitario Quirón Sur Salud, 28922 Madrid, Spain; emilio.maseda@quironsalud.es

**Keywords:** Antimicrobial Stewardship, sparing carbapenems, new antibiotics, antimicrobial resistance, individualization, antibiotic treatment, sepsis, septic shock, Gram negatives

## Abstract

The main recent change observed in the field of critical patient infection has been universal awareness of the need to make better use of antimicrobials, especially for the most serious cases, beyond the application of simple and effective formulas or rigid protocols. The increase in resistant microorganisms, the quantitative increase in major surgeries and interventional procedures in the highest risk patients, and the appearance of a significant number of new antibiotics in recent years (some very specifically directed against certain mechanisms of resistance and others with a broader spectrum of applications) have led us to shift our questions from “what to deal with” to “how to treat”. There has been controversy about how best to approach antibiotic treatment of complex cases of sepsis. The individualized and adjusted dosage, the moment of its administration, the objective, and the selection of the regimen are pointed out as factors of special relevance in a critically ill patient where the frequency of resistant microorganisms, especially among the Enterobacterales group, and the emergence of multiple and diverse antibiotic treatment alternatives have made the appropriate choice of antibiotic treatment more complex, requiring a constant updating of knowledge and the creation of multidisciplinary teams to confront new infections that are difficult to treat. In this article, we have reviewed the phenomenon of the emergence of resistance to antibacterials and we have tried to share some of the ideas, such as stewardship, sparing carbapenems, and organizational, microbiological, pharmacological, and knowledge tools, that we have considered most useful and effective for individualized decision making that takes into account the current context of multidrug resistance. The greatest challenge, therefore, of decision making in this context lies in determining an effective, optimal, and balanced empirical antibiotic treatment.

## 1. Introduction

The main recent change observed in the field of critical patient infection has been the universal awareness of the need to make better use of antimicrobials, especially in the most seriously ill patients, beyond the application of simple formulas and rigid protocols. The increase in resistant microorganisms and the quantitative increase in major surgeries and interventional procedures in the most high-risk patients, together with the appearance of a significant number of new antibiotics in recent years, have led us to shift our questions from “what to treat?” to “how to treat?” [1].

There is some controversy regarding how best to approach antibiotic treatment in these cases. Individualized and adjusted dosing, the timing of its administration, and the selection of the regimen are pointed out as factors of importance in outcomes in critically ill patients [2]. The frequency of resistant microorganisms, especially among the Enterobacteral group, together with the appearance of multiple and diverse antibiotic treatment alternatives have made the appropriate choice of treatment more complex, demanding a constant updating of knowledge and the creation of multidisciplinary teams to confront new infections that are difficult to treat [3].

In this article, we will review the phenomenon of the emergence of antibacterial resistance and try to share some of the ideas and organizational, microbiological, pharmacological, and knowledge tools that we consider most useful and effective for individualized decision making that considers the current context of multidrug resistance. The greatest challenge, therefore, of decision making in this context lies in determining an effective, optimal, and balanced empirical antibiotic treatment.

## 2. The Current Problem of Antimicrobial Resistance

Antimicrobial resistance is currently one of the greatest challenges in the treatment of critically ill patients. One of the main causes of antimicrobial resistance is the irresponsible use and overuse of antibiotics in livestock, agriculture, the food system, and the health system. This conclusion has been promoted by the One Health initiative created by the World Health Organization (WHO) (https://www.who.int/europe/initiatives/one-health (accessed on 15 January 2024)), in which antimicrobial resistance is considered the most relevant public health emergency [4,5]. In 2019, the number of deaths associated with antimicrobial resistance was estimated to be 4.95 million globally, of which a total of 1.27 million were directly attributed to antimicrobial resistance, and it is estimated that 10 million deaths could be reached by 2050. While the incidence of antimicrobial resistance among Gram-positive bacteria (GPB) appears to be stabilizing, or even decreasing, according to a recent study conducted on patients with bacteremia, the incidence of resistance to Gram-negative bacteria (GNB) is increasing and is currently responsible for the majority of cases and deaths attributable to antimicrobial resistance [6]. 

However, the fact that the existence of an infection caused by a multidrug-resistant bacterium increases mortality is controversial. The effect of multidrug resistance on costs and length of hospital stay seems clear, but a strict causal effect on mortality, despite a clear association, is debatable. The existence of uncontrolled confounding factors may have led to the determination of such an association in published studies. Among these factors, we can highlight that although multidrug resistance can be accompanied by therapeutic failure, in some cases there is no such failure. However, the use of higher doses and longer treatment times is associated with the appearance of more adverse effects, and there is also a higher prevalence of colonization and infections by multidrug-resistant patients in elderly, frail, and immunocompromised patients, which can lead to more treatment limitation decisions in intensive care units (ICUs) [7].

These pieces of data make antimicrobial resistance a major problem that requires a coordinated global action plan. The WHO has established a Global Action Plan for the Management of Antibiotic Resistance (GAP-AMR) and the creation of a Global Antimicrobial Resistance and Use Surveillance System to achieve the objectives of the program. 

Among the superbugs that we have been facing for some years now, the current concern, as previously mentioned, is focused on resistant GNB grouped together with some problematic GPB under the acronym ESKAPE: vancomycin-resistant enterococcus, methicillin-resistant *Staphylococcus aureus* (MRSA), ESBL-producing and carbapenem-resistant Enterobacterales (represented in the acronym by *K. pneumoniae*, although *E. coli,* due to its frequency of occurrence, continues to be a determining factor in mortality), *Acinetobacter baumannii,* Pseudomonas aeruginosa, and *Enterobacter* spp. [8,9]. 

Therefore, globally, we face the challenge of treating superbugs, and their prevalence within ICUs is difficult to establish [9]. To standardize the terminology, in 2012, multidrug resistance (MDR) was defined as acquired nonsusceptibility to at least one drug in three or more categories, extensive resistance was defined as nonsusceptibility to at least one drug in all but two or fewer categories, and pan resistance was defined as nonsusceptibility to all agents in all categories [10]. Risk factors for multidrug resistance often coexist in critically ill patients and even more so in the perioperative context, where the host response may be devalued by impaired depressed immune function, acute disease, or underlying comorbidities [11].

The main individual risk factors are previous exposure to antibiotics in the last 90 days, hospitalization in the previous 3 months, prolonged current hospitalization greater than 5 days, the presence of invasive devices, and previous colonization or infection by multidrug-resistant drugs. A local epidemiology with high rates of multidrug resistance is an important risk factor that needs to be taken into account [12]. The severity of the illness as a risk factor for multidrug resistance is controversial although the presence of septic shock is a recognized risk factor for multidrug-resistant pneumonia in nosocomial pneumonia and ventilator-associated pneumonia in both European and American guidelines [13]. In a recent multicenter study that included 2621 critically ill patients with intra-abdominal infection, there were no differences in the incidence of multidrug resistance between groups of patients with infections with or without sepsis and those with septic shock [14]. 

Strategies aimed at controlling antimicrobial resistance can be divided into five types:

1. Nosocomial and socio-sanitary infection prevention programs and community infection prevention programs based on hygiene and water management;
2. Vaccination as a method of preventing infection and limiting the use of antibiotics;
3. Reduction in the use of antibiotics for non-medical use;
4. Increased investment for the development of new antibiotics;
5. Minimizing the use of antibiotics for the treatment of infections in humans when they are not needed. It is this last point that has been promoted by the Antimicrobial Stewardship (AMS) concept.

## 3. Antimicrobial Stewardship (AMS)

The goal of AMS is the correct and effective administration of antibiotics, thus minimizing the emergence of resistance, ensuring sustainable use by optimizing the use of antimicrobials in empirical treatment, and refining targeted treatment [15]. However, in a broad sense, what this type of program should pursue is the creation of a “culture of infection” led by a few people who exercise such leadership in a non-coercive way and who decisively influence the rapid diagnosis and correct prescription of antimicrobials based on continuous training and equipment. With a multidisciplinary operating philosophy, this program is not limited to the creation of protocols or to the members of the unit. The existing recommendations regarding who should be part of the responsible group advise that the group should be made up of experts or those more specialized in infection within the ICU, microbiologists with a special vocation for the critically ill patient, and, optionally and according to the hospital’s model, pharmacologists and doctors with special dedication to infection regardless of their specialty [16].

In short, AMS programs thus acquire the meaning of “taking responsibility for what one is not directly responsible for” and have been shown to improve the outcomes of critically ill patients with sepsis, reduce the emergence of resistance in ICUs and in the hospital, significantly reduce errors and side effects, and control, in the best sense, costs [17,18,19].

The European Society of Intensive Care (ESICM) has created a very interesting educational platform led by Jan de Waele and Maurizio Cecconi for training in the creation and development of AMS (https://www.esicm.org/education/educational-initiatives/quality-improvement-antimicrobial-stewardship/ (accessed on 15 January 2024)). 

The implementation of an AMS program begins by identifying the barriers that prevent an adequate management of infection in a process of continuous improvement and seeking alliances with microbiology services, sepsis code groups, and others. 

In this sense, sepsis code programs (performance improvement programs in sepsis) have proven their effectiveness in reducing mortality and are another example of the importance of the “how” over the “why” [20,21]. They have been encouraged by the guidelines of the Surviving Sepsis Campaign (SSC) [22] and include a set of clinical, organizational, analytical, and microbiological tools that, together with intense training and cognitive aids, have the mission of improving the outcomes of patients with sepsis, prioritizing their care and refining their treatment [23]. These programs’ focus is limited to the first 24–36 h of care for patients with sepsis and septic shock, and their philosophy includes optimization and continuous improvement in empirical antibiotic treatment. There are more and more alliances between these types of models and AMS programs since the patients with the most pressing antibiotic prescriptions are patients with sepsis, and they require frequent re-evaluations, after the first 36–48 h, to de-escalate, escalate, or change their treatments.

To this end, it is essential to know the local resistance patterns using local guidelines and those published by experts and scientific societies as cognitive aids in order to have solid knowledge of microbiology that allows communication with the experts in our hospital and that allows us to improve the pharmacokinetic aspects of administration, to consider possible drug allergies, and to promote educational initiatives aimed at a better use of antibiotics. In the following sections, we will present some of these initiatives, review the most useful classic concepts, and suggest other more innovative and avant-garde aspects [16].

### 3.1. Start Smart

This model of approach to severe infection in critically ill patients encompasses both the optimization of diagnosis and the form of administration and de-escalation strategies, all under the umbrella of AMS programs. In this context, the existence of new biomarkers and rapid microbiological techniques is a key element in this new approach.

### 3.2. Start Smart and then Focus

A few years ago, the government of the United Kingdom promoted the slogan “*Start Smart and then focus*” (https://www.gov.uk/government/publications/antimicrobial-stewardship-start-smart-then-focus) (accessed on 17 January 2024), and we believe that this slogan can be a good model for an approach to the antibiotic treatment of sepsis since the intelligent administration of antibiotics is closely related to a personalized approach, which does not deal with the treatment of all very serious cases in the same way, e.g., a patient in a situation of shock and with a high bacterial load (in which the slogan is “*hit fast and strong!*”) will not be treated in the same manner as a less severe case who presents a moderate suspicion of infection, even when both may be admitted to the ICU for similar reasons [24].

Key takeaways from a smart start to antimicrobial therapy include

- Only start antimicrobial therapy if there is clear suspicion or evidence of infection;
- Have a complete history of drug allergies and other patient considerations;
- Initiate early and effective treatment in patients with sepsis and/or life-threatening infections;
- Avoid the widespread use of broad-spectrum antibiotics, without paying attention to accompanying clinical symptoms, when treating any suspected infection;
- Be up-to-date and always keep in mind the local microbiology and the patterns of resistance prevalent in the unit, the hospital, and the environment;
- Obtain cultures before starting therapy when possible, taking into account that treatment should not be too greatly delayed;
- In critically ill patients with septic shock, use the best antimicrobial alternative available to resolve the infection, in addition to controlling the outbreak whenever feasible, to reduce the bacterial load as soon as possible [24].

Finally, and as one of the clearest and firmest precepts of “start smart”, it is necessary to ensure that all decisions related to infection are documented in the medical record because, very often, treatments are maintained unnecessarily because clinicians do not have the information about the reason for starting these antibiotics nor when they were started and how long it was planned to continue with them. The situation is aggravated when a patient’s care is passed from one doctor to another in successive work shifts. Therefore, it is recommended to include in the record the name of the antibiotic and its dose, the reason for the indication, the severity of the disease, the estimated duration of treatment, and the next review date. 

### 3.3. “… And Then Focus”: What Does This Consist of

The second part of this motto reminds us of the imperative need to review the clinical diagnosis and consider the possibility of changing, de-escalating, or even escalating treatment. Thanks to current advances in rapid microbiological diagnosis, this review may be possible in less than 48 h, allowing decisions to be made about the need to continue antibiotic treatment with the same or other antibiotics, and at what dose, and to document a clear plan of action on the decision to prescribe these antibiotics [25]. Every day, it is necessary to question whether the antibiotic is adequate, whether the dose is optimal, and whether the patient needs it [26]. 

The Challenge of Treating Superbugs: What to Think About if Antibiotic Treatment Fails

- Delay in administration;
- Inappropriate spectrum of activity;
- Inadequate blood levels or inadequate penetration into the focus of the infection;
- Presence of antibiotic neutralizers and/or antagonists;
- Presence of superbugs;
- Presence of a superinfection;
- Non-infectious source of fever;
- The focus of the infection has not been properly controlled.

## 4. Microbiology: Key Concepts in Sepsis

The role of microbiology in sepsis is key and requires clinicians to have solid microbiological notions that allow them to correctly understand information. We will briefly review some points of knowledge that we consider essential in their daily application to achieve an improvement in results [27,28].

### 4.1. Specimens and Diagnostic Techniques

A good microbiological diagnosis must begin with a good choice of the sample provided to the laboratory; this sample should be representative of the suspected infection and its processing should observe correct standards of collection, shipment, documentation, and storage. Microbiological culture continues to be the reference method as it not only makes it possible to characterize the germs causing the infection but also allows the relevant antimicrobial susceptibility tests to be carried out on the sample. However, the need to obtain results quickly and technological advances have led to the development of molecular techniques that are revolutionizing microbiological diagnostics [29]. 

Among the rapid techniques, two currently stand out:

- MALDI-TOF (matrix-assisted laser desorption ionization–time of flight): a technique based on mass spectrometry. MALDI-TOF currently allows the identification of microorganisms in less than 30 min (previously, it took up to 18 h) through the analysis of their protein profile. It is perhaps the most widespread rapid technique [30].
- RT-PCR (real-time polymerase chain reaction): a molecular technique that allows the direct identification of germs from the sample.

Other molecular techniques available seem to be less widespread today due to their high cost [30], and point-of-care *(POC)* rapid microbiological diagnostic systems are becoming increasingly common in ICUs [31].

### 4.2. Minimum Inhibitory Concentration (MIC)

When performing an in vitro antibiotic susceptibility technique, the main objective is to predict whether a patient with an infection by a certain germ can be treated with an antimicrobial or if another option should be chosen.

Minimum inhibitory concentration (MIC) is defined as the lowest concentration of an antimicrobial that inhibits the growth of a microorganism after incubation. MIC, therefore, is crucial to be able to confirm the resistance of microorganisms to an antimicrobial agent as well as to monitor the activity of new antimicrobial agents [32]. 

Classically, we know that there are both time-dependent and concentration-dependent antibiotics. Time-dependent antibiotics are considered adequate to treat an infection caused by a bacterium when, administered in doses considered therapeutic (i.e., non-toxic), they reach a plasma concentration at the site of infection at least four times higher than the MIC most of the time. Concentration-dependent antibiotics are those that are considered adequate to treat an infection when they reach a peak concentration 10–12 times above the MIC value.

For the interpretation of susceptibility tests, the European Committee on Antimicrobial Susceptibility Testing (EUCAST) defined the following categories since 2019 [33]:

S: sensitive. Thus, a bacterium that is inhibited in vitro by a concentration of the antimicrobial is associated with a high probability of therapeutic success.

I: (formerly intermediate) sensitive with increasing exposure. In this case, the microorganism is sensitive when exposure to the antibiotic is increased; therefore, there is a high probability of therapeutic success because exposure to the agent is increased by adjustment of the dosage regimen or by its concentration at the site of infection. 

A: resistant. The treatment of the microorganism studied for the given antibiotic has a high probability of therapeutic failure.

To include an antimicrobial in one of these categories, a breakpoint (BP) is required, which is the MIC that determines inclusion in a specific category. To define these BPs, it is necessary to carry out a prior study of various parameters, including the officially approved dosage, the microbiological data of each germ, pharmacokinetic and pharmacodynamic data, as well as certain relevant clinical data, such as the place where the sample was obtained.

There are different national and international bodies that carry out studies to define BP (Clinical Laboratory Standard Institute, EUCAST, etc.). Currently, EUCAST determines the PRs in Europe and publishes their data on an open-access website: http://www.eucast.org (accessed on 17 January 2024). As microbiological, pharmacokinetic, pharmacodynamic, and clinical knowledge advances, the EUCAST periodically reviews the BPs and is able to observe that a previously sensitive microorganism, presenting the same MIC, can be defined a year later as having increased sensitivity or even as resistant. 

MIC is not only used to determine the concentration of antimicrobial that the patient will receive but also the type of antimicrobial to be used, allowing for reduction in the opportunity for microbial resistance to specific antimicrobial agents to develop. This does not mean that it is necessary to choose antimicrobials that have a lower MIC value, as other factors, such as the pharmacokinetics of the antimicrobial and the concentration reached at the site of infection, play a role. However, a lower MIC value induces a higher probability of achieving the therapeutic target and the possibility of a lower development of toxicity and resistance mechanisms, especially mutational ones. It is important to note that MIC is defined for each microorganism with respect to each antibiotic; therefore, strictly speaking, the fact that one antibiotic has a lower MIC than another for the same microorganism does not have therapeutic implications. On the other hand, referring to the same antibiotic for a certain microorganism (e.g., an MIC of 1 μg/mL or 8 μg/mL), despite both possessing “sensitive” MIC values, will have therapeutic implications. Continuing with the example, with an MIC of 8 μg/mL, a higher dose of antibiotic will be necessary to achieve the optimal treatment, which may increase toxicity or imply the need for an extended infusion treatment to assess a second sensitive antimicrobial to make synergy or, in some cases, it will induce the decision to use another antibiotic despite this other antibiotic having been classified as “sensitive”, as previously mentioned [33]. 

### 4.3. Preventive Concentration of Mutants

Mutant preventive concentration (MPC) is the MIC of the least sensitive mutant among a heterogeneous bacterial population and represents the lowest antibiotic concentration that prevents or blocks the emergence of resistant mutants [34]. 

There is a window of concentrations that prevents the emergence of resistant mutants. The exposure of a bacterial population to the action of an antimicrobial usually produces a deleterious effect on it, inhibiting its growth or causing its death. This effect is not always obvious, either due to the presence of a previous resistance mechanism in it or due to the emergence of resistant mutants. During antimicrobial administration, selection of resistant mutants can occur dynamically over time. This is related to the dose of the antimicrobial and its frequency, its pharmacokinetics at the site of infection, and the time during which concentrations equal to or greater than the MPC are reached. Antimicrobials can be described as having a mutagenic effect and inducing mutations. However, resistant mutants are found naturally in bacterial populations independently of the presence of the antimicrobial in the environment. Every bacterial population has, with varying frequency, resistant mutants that coexist with the most susceptible population. Under certain circumstances, when a bacterial population is subjected to the action of an antimicrobial, resistant mutants can become dominant by inhibiting the most sensitive fraction, but not that of resistant mutants. This process is known as *resistant mutant selection*. In these cases, the probability of selection of these mutants would be higher with inappropriate treatments not adjusted to pharmacokinetic/pharmacodynamic parameters (Pk/Pd). The possibility of entering the selection window during treatment would be higher, especially with inadequate dosages [35].

MPC is not strictly a parameter of antimicrobial activity, which would be better determined by the value of the MIC and its correct interpretation; however, it could be used as such. Its application to the ability to restrict or decrease the selection of resistant mutants when antimicrobials are used with Pk/Pd criteria and, therefore, to the possibility of avoiding associated clinical failure would lead to the assumption that MPC is an activity parameter. Likewise, the concept has a clear clinical application and helps to define the most appropriate dose of an antimicrobial, at least from the point of view of mutant selection, and differentiates between the antimicrobials that should be used in the development of treatment guidelines and definition of antimicrobial policies [36].

### 4.4. The Importance of Rectal Exudate

Within the microbiological tests in critically ill patients, we highlight the role that rectal exudate can play as an effective and high-yield surveillance culture that can determine which microorganism is potentially causing a serious infection and how to act based on the results. The result of this test, available in 24–36 h, can be very useful, when the causative microorganism is not identified, to help one to decide what to designate as the definitive empiric antibiotic treatment. 

Most nosocomial infections are endogenous and originate in the mucosal microbiota. This endogenous infection occurs due to translocation of the predominant aerobic microorganisms in the intestinal mucosa, and the likelihood of its diagnosis depends on its density and colonized area. The significant or repeated presence of a GNB in the rectal exudate implies its overgrowth in the intestinal lumen, and the relationship between intestinal colonization and the risk of bacteremia, infection by carbapenemase-producing microorganisms, or candidemia, among others has been verified [37]. The composition of the gut microbiota can change within 72 h of the arrival of a new microorganism or the start of antibiotic treatment; thus, determining the microorganisms that make up that microbiota and their sensitivities provides us important indices concerning the possible causes of infections.

For this reason, it is recommended to actively search for the presence of MDR in all patients by obtaining rectal exudate, depending on the system, regardless of whether other cultures are obtained. This is especially true in patients with septic shock at the time of admission to the ICU. This process should be carried out at least once a week throughout their stay [24].

Likewise, upon admission of a patient to the ICU, a “checklist” should be completed in order to identify those at high risk of being carriers of MDR, especially those resistant to carbapenems [38]. The checklist should be based on 14 different studies that have identified risk factors most often associated with the possibility of an individual being a carrier of MDR:

1. More than 5 days of hospital admission in the previous 3 months;
2. Institutionalized patients;
3. Known MDR colonization or infection;
4. A duration of more than 1 week of antibiotic treatment in the previous 3 months (especially with third- and fourth-generation cephalosporins, quinolones, or carbapenems);
5. Patients with chronic renal failure undergoing dialysis;
6. Patients with chronic pathology and high incidence of MDR infections (bronchiectasis, cystic fibrosis, etc.).

## 5. Pharmacokinetics and Pharmacodynamics of Beta-Lactams

Given the scarcity of effective drugs against new strains of resistant drugs, much more attention has been paid to Pk/Pd, and Pk/Pd has been granted the importance it deserves when choosing antibiotics and how they are administered [39]. 

Due to their importance and frequency of use, we will focus on the study of the Pk/Pd of beta-lactams. These drugs are time-dependent: their antibacterial efficacy is related to a parameter that contemplates the time when their plasma concentrations are between four and eight times above the MIC. According to experimental studies and depending on the stability of each commercial form, it is recommended to administer these drugs in an extended infusion of between 1 and 4 h or even in continuous infusion, at least when MDR treatment is considered and is possible due to the stability of the drug.

Although the expected clinical efficacy of this proposal has not been demonstrated, either due to an absence of effect, insufficient sizes in the comparative studies carried out, or even due to possible methodological errors, studies as influential as DALI that demonstrated insufficient exposure [40] and the influence of authorities and societies have made prolonged infusions of beta-lactams a standard in the ICU [41]. 

In this regard, it is interesting to comment on the results of a recent randomized controlled trial (RCT) with apparently contradictory results on this topic. Regarding meropenem, to date, the administration of a total daily fractionated dose was recommended, but in an extended infusion of 3–4 h. The RCT “MERCY Trial” has shown that in critically ill patients with sepsis or septic shock, there were no differences in mortality at 28 days or in the appearance of resistance between continuous infusion administration of the same dose of 3 g in 24 h and intermittent administration (1 g every 8 h, administered over 30–60 min). As indicated in the editorial accompanying the study, since infusions do not cause harm or entail an additional cost, it is difficult for the guidelines to discuss even the published meta-analyses that indicate a possible decrease in mortality [42]. In fact, the current consensus recommendations of several societies, while acknowledging a low level of evidence, continue to suggest the use of prolonged-infusion (PI) beta-lactams versus short-infusion (SI) beta-lactams in adult patients with severe disease, particularly in cases of GNB infection [42].

Furthermore, in this sense, there has been interest in the administration of antibiotics through routes other than intravenous for the treatment of certain infections. This is the case for aerosolization for the prevention and treatment of respiratory infections related to intubation, usually associated with intravenous administration. However, the lack of sufficient RCTs [43], together with the need for specific devices for proper aerosolization, means that aerosolization remains a practice not recommended by the American Thoracic Society (ATS), the Centers for Disease Control and Prevention (CDC), or the European Society of Clinical and Infectious Diseases (ESCMID) [44,45,46]. However, in clinical practice, the indications must be discussed and personalized for each case. Although there is no quality clinical evidence, there is a large amount of experimental evidence that in frail patients with severe pneumonia caused by MDR bacteria and at high risk of treatment failure, nebulized antibiotic therapy may increase the possibility of reaching very high concentrations of the drug at the site of infection to facilitate therapeutic success, associated with improved intravenous therapy [47]. 

Although there is still a lack of evidence for the new beta-lactams with beta-lactamase inhibitor (BL-BLI), therapeutic drug monitoring (TDM) can solve certain problems if we also remember that it is possible that any antibiotic has the risk of reaching “insufficiently stable” plasma concentrations during continuous infusion administration. Although PI administration or extended infusion may reduce this risk somewhat (since it will at least be easier to achieve sufficient plasma peaks during administration), only TDM can confirm the achievement of the desired levels [48]. 

In the absence of this, and thanks to the wide therapeutic margin of beta-lactams, in critically ill patients with high volumes of distribution and a possible increase in clearance, we believe that it is preferable to administer the highest recommended doses rather than favoring an administration in lower doses [49]. 

## 6. Allergy to Beta-Lactams

The label of allergy to beta-lactams is often self-imposed by the patients themselves, and, in a large number of cases, this label is applied due to the appearance of symptoms that do not even possess an underlying immune mechanism (diarrhea, headache, etc.). It is estimated that this label has been applied to 15% of the general population and is true in less than 10% of these cases (therefore, only in approximately 1% of the population) [50,51]. For this reason, it is important to lay certain foundations that are increasingly necessary so as not to limit the use of an antibiotic group that is the first line of treatment in many infections due to its more or less broad spectrum and because it is inexpensive and generates few side effects for the patient.

Strictly speaking, only immunological reactions mediated by IgE (type I of the Gell and Coombs classification) are considered true allergies. The presentation of these reactions is varied and, generally, appear in the first hour following administration of the drug. Although not all of these reactions are severe, the most severe forms (anaphylaxis and bronchoconstriction) are life threatening and contraindicate subsequent administration of the drug that causes them. However, there are other types of immunological reactions mediated by other different and immunocomplex antibodies (type II and III reactions) or by T lymphocytes (type IV reactions) that either contraindicate such administration, such as severe skin syndromes, or may require caution and follow-up in successive administrations (such as cytopenia).

We now know that beta-lactams generate few cross-reactions between them due to the beta-lactam ring itself, and most cross-reactions are due to the similarity of the side chains that are shared between various drugs in the group, which makes such cross-reactions more frequent between certain beta-lactam compounds (for example, between aminopenicillins and aminocephalosporins) but not between all of them [52].

The drug challenge or oral test is still the gold standard of diagnosis. However, it is notable that scientific societies generally consider screening through antecedents and clinical history to be insufficient [53,54], while many authors require its use in emergency situations and propose an approach that allows safe and immediate administration in severe cases where there are no plausible alternatives [55,56,57,58].

## 7. What Should I Know about Old Antibiotics

An interesting study carried out by the ESCMID Study Group for Antibiotic Policies in several European countries, the USA, Canada, and Australia showed that drugs such as cloxacillin or aztreonam were only available in about half of these countries, that not all of them had colistin, and that only 5 out of a total of 38 possessed colistin for the intravenous formulation of fosfomycin [59]. However, the emergence of resistance, especially among GNB, has revived interest in the use of this type of drug with good activity against MDR germs, a long history of use, and which, according to this study, had been relegated to the background due to toxicity because the drug has a narrow spectrum or for market reasons [60].

Among these drugs demonstrating activity against GNB are fosfomycin, colistin, and cotrimoxazole. All of these have hardly any activity against anaerobes, and their activity against GPB is variable. 

Regarding fosfomycin, there seems to be consensus on its usefulness only as an alternative to nitrofurantoin or trimethoprim-sulfamethoxazole (TMP-SMX) in uncomplicated cystitis caused by GNB-producing extended-spectrum beta-lactamases (ESBLs) or carbapenemases. In renal parenchyma, it does not reach sufficient concentrations, so it is not recommended in cases of complicated urinary tract infection (UTI), bacteremia, or other serious infectious conditions with a non-urinary focus and should only be used when the causative germ is *E. coli*, since *K. pneumoniae* and other Gram-negative causes of the condition are frequent carriers of *fosA* hydrolase that inactivates the drug. However, in the context of the prevalence of MDR germs, its use has increased, especially in combination with other drugs [61]. 

Colistin, with a known nephrotoxic potential and a narrow therapeutic index, was formerly recommended in antibiotic therapy association schemes to treat carbapenemase-producing GNB or Pseudomonas aeruginosa *MDR*, but the availability of the new BL-BLI and the growing increase in resistance to colistin due to its use in recent years have contributed to this practice being advised against. Currently, colistin is practically relegated to the treatment of *Acinetobacter baumannii* infections and for those cases of resistance or intolerance to the new BL-BLI [62]. 

Cotrimoxazole plays a key role in UTIs (complicated or not), both those caused by ESBL-producing enterobacteriaceae and those resistant to carbapenems. It loses its usefulness against Pseudomonas aeruginosa but recovers it against *Stenotrophomonas maltophilia*, against which it is one of the treatments of choice [63]. However, as with colistin, the use of alternatives, such as cefiderocol or the combination of ceftazidime/avibactam with aztreonam, is infrequently indicated in our ICU [64].

Tigecycline has broad-spectrum activity that makes it an attractive option for MDR infection [65]. However, it has been reported that it can lead to treatment failure in some contexts if used as monotherapy, as it is a bacteriostatic antibiotic [66]. 

Therefore, these alternatives should not generally be recommended for current empirical use nor, in the context of septic shock, in environments with a high proportion of MDR.

In terms of GPB—although in our environment, despite the global increase in infections caused by *Staphylococcus aureus*, there is a decrease in the percentage of serious infections caused by MRSA—it should be recalled that in the United States alone, GPB are responsible for more deaths than emphysematous disease, Parkinson’s disease (antimicrobial resistance surveillance in Europe, 2021–2023 data; Stockholm: European Center for Disease Prevention and Control and World Health Organization, 2023), HIV/AIDS, and homicides combined [67]. In the treatment of these infections, it is also worth mentioning the role of TMP-SMX (a cheap and desirable drug for sequential treatment) as well as that of rifampicin and clindamycin. None of them are presented as the drug of choice compared to more effective alternatives such as vancomycin, linezolid, and daptomycin. 

TMP-SMX only seems to be recommended for infections that require a long treatment time (bacteremia and nosocomial pneumonia) and once the susceptibility of the germ to it has been demonstrated [68]. 

Rifampicin and clindamycin are only recommended as part of a combination regimen, in association with first-line treatment, in certain conditions such as osteomyelitis, necrotizing pneumonia, meningitis, and, especially, in the case of rifampicin, endocarditis on the prosthetic valve, due to the potential greater eradication effect of rifampicin on prosthetic material [69].

Finally, it is worth noting the potential role of quinupristin-dalfopristin in the alternative treatment of infections caused by *E. faecium* when this germ presents resistance to vancomycin, linezolid, and daptomycin [59].

## 8. What I Need to Know about New Antibiotics

The 10 antibiotics by 2020 initiatives offered by the Obama administration, facilitating patents on new antimicrobials, have allowed us to witness, in recent years, the emergence of new antibiotics that have improved the arsenal available for the treatment of MDRs and whose judicious use places greater value on the existence of AMS programs [70]. 

A comprehensive review of new antibiotics is beyond the scope of this review, and there are very good reviews, both specific and generic, in the literature [71,72]. For this reason, and before going into more specific recommended guidelines, we will briefly review some of the characteristics of the new antibiotics. We will expand a little more on ceftazidime/avibactam, due to the already existing clinical experience and ceftazidime/avibactam’s profile for empirical treatment in the context of multidrug resistance. In addition, more briefly, we will make some interesting considerations about ceftolozane/tazobactam and cefiderocol.

Table 1 shows the new and most important treatments already on the market or about to be marketed and some of their key characteristics, such as dose and spectrum of action.

### 8.1. Ceftazidime/Avibactam

Ceftazidime/avibactam is a combination of an existing cephalosporin, ceftazidime (inhibitor of bacterial cell wall synthesis, which causes cell death), and a new inhibitor of non-ß-lactam ß-lactamase, avibactam (inactivator of certain beta-lactamases by covalent acylation of beta-lactamases and which does not, per se, possess antibacterial properties). The addition of avibactam restores the in vitro activity of ceftazidime against a significant number of beta-lactamases, including class A enzymes of the Ambler classification (ESBL and *K. pneumoniae* carbapenemases (KPCs)), class C enzymes (Amp C), and certain class D enzymes (OXA-48 carbapenemases). It does not exhibit activity in the presence of class B enzymes (metallo-ß-lactamases) [73]. Therefore, it has the spectrum of antipseudomonic actions of ceftazidime and is active against other GNB, in general, and against Enterobacterales, in particular. However, it does not show activity against *Acinetobacter*, *Stenotrophomonas maltophilia*, or anaerobes (thus, it is worth recalling that an anaerobicide antibiotic must be associated with intra-abdominal infection). The antibiotic is registered by the Food and Drug Administration (FDA) and the European Medicines Agency (EMA) to treat complicated intra-abdominal infections, complicated urinary tract infections, hospital-acquired pneumonia, and pneumonia associated with mechanical ventilation [74]. 

The efficacy of ceftazidime/avibactam is corroborated by significant clinical evidence, and various societies and experts recommend its use as a first alternative in the targeted treatment of infections caused by carbapenemase-producing enterobacteriaceae such as OXA-48 or KPC as well as its alternative use as a single drug, or in association with other drugs, in other situations related to *Pseudomonas* infection or carbapenemases other than those mentioned above [64,75,76,77].

Real-world evidence is also indicative of favorable outcomes, even in MDR5 infections [78]. We will highlight the EZTEAM that included 569 patients in 11 countries, treated in the first or second line with ceftazidime/avibactam, with all types of severe infection, in which 89.3% were resistant to carbapenems [79]. 

Regarding the importance of administering the drug early, use within 48 h after the onset of infection has been associated with better clinical outcomes [80]. An example is the study by Jorgensen et al., which included 203 patients with MDR infections, 117 of whom were carbapenem-resistant Enterobacterales (CRE) or *Pseudomonas* [81]. Among the 203 patients, 91 received ceftazidime/avibactam within 48 h of the onset of infection. The clinical success rate when treatment was initiated within 48 h was 33.3% and failure was 18.6%. Every 24 h delay in ceftazidime/avibactam worsened outcomes significantly, whereas the regression model for clinical failure showed that ceftazidime/avibactam administered within 48 h of culture collection was protective (OR: 0.409). 

Our group participated in another interesting study to assess the possible advantages of ceftazidime/avibactam in empirical treatment [82]. In this retrospective study of 339 patients, treatment with ceftazidime/avibactam in OXA-48 and KPC carbapenemase-producing infections was compared with the best available therapy that included carbapenems with tigecycline, colistin, or fosfomycin, among others. We observed that treatment with ceftazidime/avibactam was associated with longer survival. Interestingly, the most severely ill patients had better outcomes with ceftazidime/avibactam than with monotherapy. 

Studies like these, along with other real-life studies, highlight the advantages of using ceftazidime/avibactam in an early stage of sepsis and for the most severe patients. For this reason, the Spanish consensus assesses severity with the Charlson scale, among others, to make decisions on empirical antibiotic therapy and includes ceftazidime/avibactam as one of the main antibiotics in empirical treatment, similar to meropenem, given ceftazidime/avibactam’s efficacy in these MDR scenarios, its very broad spectrum of action with respect to MDR GNB, and its bactericidal and synergy capacity [24]. 

Regarding synergies, it is very interesting to analyze these in the context of nosocomial infection in units with a high number of MDRs. The synergies of ceftazidime/avibactam with meropenem, amikacin, aztreonam, colistin, or fosfomycin against multidrug-resistant *Klebsiella pneumoniae* and Pseudomonas aeruginosa have been studied, resulting in beneficial association, significantly reducing the bacterial load in logarithmic form and, in the case of meropenem and aztreonam, restoring their activity against these microorganisms [83]. Therefore, the association of ceftazidime/avibactam and meropenem could make sense in the presence of MDR microorganisms in patients with septic shock and high bacterial load. 

### 8.2. Ceftolozane/Tazobactam

Ceftolozane/tazobactam is a new antibiotic composed of a new cephalosporin associated with tazobactam, a known β-lactamase inhibitor. Ceftolozane/tazobactam is active against Pseudomonas aeruginosa MDR bacteria, including those resistant to carbapenems, and against ESBL-producing enterobacteriaceae. However, it is not active in the case of CRE. The drug is approved by the FDA and EMA for the treatment of intra-abdominal infections, complicated urinary tract infections, and hospital-acquired and ventilator-associated bacterial pneumonia [84]. 

The lack of activity in the face of CRE limits ceftolozane/tazobactam’s empirical use in some scenarios. However, when the focus of the suspicion of sepsis is nosocomial pneumonia, its empirical results are very good, and it can be recommended as an empirical treatment in this situation [85]. Of particular note is the study by Timsit, a sub-study of the pivotal trial for the indication of pneumonia of ceftolozane/tazobactam (ASPECT), which compared the efficacy, in terms of mortality, between a 3 g dose of ceftolozane/tazobactam and meropenem against hospital-acquired pneumonia and ventilator-associated pneumonia. The outcomes were significantly better for patients treated with ceftolozane/tazobactam, especially in the group of pneumonia requiring mechanical ventilation [86]. This is important, considering that patients with hospital-acquired pneumonia who progress to respiratory failure severe enough to require mechanical ventilation are considered to represent the most clinically severe subtype with the worst outcomes.

Its real-life use in critically ill patients has also been validated in various types of infections, especially in the context of *Pseudomonas* infection [87]. 

### 8.3. Cefiderocol 

Cefiderocol is a catechol-type cephalosporin siderophore with very potent in vitro activity against drug-resistant CRE and non-fermenting GNB. It uses the siderophore–iron complex pathway to penetrate Gram-negative membranes through a mechanism that has been compared to the well-known Trojan horse. Once inside the bacterium, cefiderocol separates from iron and binds to penicillin-binding proteins to inhibit peptidoglycan synthesis [88]. This antibiotic appears to be more stable against the hydrolysis of many ß-lactamases and carbapenemases. Its spectrum of action—in principle, the broadest of the antibiotics marketed for GNB, including multidrug-resistant antibiotics and especially metallo-beta-lactamase producers—has led to high expectations for this drug [89]. However, in the ambitious phase 3 CREDIBLE-CR20 study comparing cefiderocol against the best available treatment (chosen by the investigator, with a combination of up to three drugs) for the treatment of severe infections caused by GNB MDR and CRE, the results were as follows: similar clinical cure rates were obtained in both groups and mortality was numerically higher in the subgroup of patients with *Acinetobacter baumannii* infection treated with cefiderocol. However, this subgroup had a selection bias since these patients were more frequently in shock in a significant way, bringing into question whether, for this reason, the results were worse [90]. 

The antibiotic has been approved by the FDA and EMA for GNB infections when other treatments might not work, and, in real life, the results with cefiderocol are very effective against *Acinetobacter*-, *Stenotrophomonas-*, *Pseudomonas-*, and Enterobacterales-producing carbapenemases [91].

## 9. Cutting-Edge Approaches to New Antibiotics

### 9.1. Sparing Carbapenems

The concept of sparing carbapenem aims to save this type of antibiotic and judiciously use new drugs that can participate in this strategy, keeping in mind the appropriate infection control and prevention measures within stewardship strategies [92]. 

Carbapenems are excellent antibiotics and a powerful weapon against drug-resistant Gram-negative bacteria because of their broad spectrum, their bactericidal effect, and their ability to circumvent many resistances. Their excessive use, in the absence of safe alternatives to date, especially in empirical treatment when there is a risk of MDR, has generated a problem of resistance to carbapenems, especially carbapenemases [93]. 

Intestinal colonization is the main reservoir of carbapenem-resistant GNB (CRE) among critically ill patients, and intestinal translocation is associated with an increased risk of developing CRE in successive infections. Not surprisingly, overuse of carbapenems is one of the critical determinants for CRE acquisition and is associated with a higher rate of superinfection compared to non-carbapenem treatments. With carbapenem treatment, there is an increased risk of developing resistance due to the pressure carbapenems exert on sensitive strains, providing resistant strains the opportunity to overcome them [94]. 

Among the strategies used to spare carbapenems, we will highlight the following [3]:

1. Assessing the indication for the use of carbapenems when there is no clear risk of MDR;
2. Establishing in which patients piperacillin tazobactam may still play an important role, even with the negative results of the MERINO trial [95];
3. Assessing the use of new beta-lactam antibiotics and new beta-lactamase inhibitors with which there is already significant clinical experience as an alternative to carbapenems in the empirical treatment of patients in septic shock, especially if there is a risk of MDR in the context of ESBL and CRE [77];
4. Assessing the combined use of alternatives based on non-beta-lactam drugs and old antibiotics in some contexts [96];
5. Adjusting the duration of therapy with carbapenems. Recent data indicate that short-term treatments (<10 days) for Enterobacterales do not worsen clinical outcomes [97];
6. Assessing the role of de-escalation following a rigorous microbiological control associated with a clinical assessment within stewardship to reduce the need for maintenance over time during empirical therapies with carbapenems [98].

Preserving carbapenems—though carbapenems should still occupy a central place in the antimicrobial therapy of critically ill patients—is vital for success in improving outcomes in septic shock. The use of the new beta-lactams and beta-lactam inhibitors in stewardship and de-escalation programs, taking into account the desire to spare carbapenems, constitutes the best course of action in the present and, hopefully, the future, especially in the contexts of the prevalence of difficult-to-treat pathogens such as ESBL and CRE and resistance to *Pseudomonas* and *Acinetobacter* (Table 2) [27].

### 9.2. Role of New Antibiotics in the Empirical Treatment of Nosocomial Sepsis and Septic Shock

Undoubtedly, the most difficult and controversial issues in the treatment of sepsis include determining a personalized empirical antibiotic treatment and defining the role played by new antibiotics in this empirical regimen [99]. 

Although targeted therapy against GNB is not without controversy either, today we have several guidelines for targeted treatment against MDR germs (producers of ESBL and/or carbapenems, difficult-to-treat *Pseudomonas*, or other problematic germs such as *Acinetobacter* or *Stenotrophomonas*). These guidelines are very balanced and indicative and have a very similar background, often based on up-to-date expert opinion, due to the rapid emergence of new drugs and published evidence, or using a GRADE methodology [64,75,76]. Beyond differences related to the positioning of particular drugs due to contradictory trials and the role played in previous years by old antibiotics used for MDRs, antibiotics are very clearly positioned, particularly those aforementioned drugs that have been the subject of numerous publications and consistent clinical experience, such as ceftazidime/avibactam or ceftolozane/tazobactam. Meanwhile, newer molecules, such as cefiderocol or carbapenems associated with beta-lactamase inhibitors, are treated conservatively as we await real-life results. Together with specific guidelines and consensus on the treatment of the main infectious syndromes associated with sepsis (hospital-acquired pneumonia, ventilator-associated pneumonia, secondary and tertiary peritonitis, bacteremia associated with other infections, or urinary tract infection), these newer molecules can provide very important cognitive aid when deciding on empirical therapy within an AMS policy in scenarios of high severity and risk of death. 

Before entering a discussion that specifically centers on empirical antibiotic treatment in patients with septic shock of nosocomial origin, we must contextualize this with the available evidence. This is a field in which there are several theoretical models, all supported by evidence but not all of the same quality, that are defended by legitimate and elaborate rationales. We can divide these models into two categories determined by the two consensuses referred to above. One is the more conservative French model, which advocates reserving the use of new antibiotics in empirical treatment, and even not using them, under the aegis of sparing carbapenems [100]. The other model is exemplified by the Spanish consensus, which advocates the empirical use of new antibiotics for their efficacy against MDR in the context of the pressing need to apply a correct treatment the first time, especially in the presence of risk factors related to the infection and patient [24]. In this area, the balance oscillates between increasing the probability of employing the correct empirical treatment and the generation of resistance associated with an overuse of the new antibiotics. However, there are some common denominators in the literature and, specifically, between these two French and Spanish consensus guides [24,100]:

1. In the case of septic shock, the use of new antibiotics is accepted in the empirical study, especially in cases where there is a high local proportion of MDR and in those carrying MDR germs in epidemiological surveillance cultures, especially if this is rectal exudate;
2. Strategies such as rapid microbiological diagnosis, de-escalation, and shortening of the duration of antibiotic treatment are strongly recommended in order to avoid the side effects associated with overuse of antibiotics, regardless of the broad-spectrum regimen chosen.

It seems to be demonstrated that there is an overuse of antibiotics in this scenario of intensive care and sepsis, since there is a significant percentage of patients for whom broad-spectrum antibiotics are prescribed and infection is not demonstrated. In addition, empirical guidelines are often maintained over time, either due to the lack of rapid tests or the absence of an AMS that questions treatments on a daily basis [94]. 

This overuse has its consequences. We will point out some of the main ones or those in which there is currently the greatest interest:

1. Affecting the patient’s microbiota, selecting MDR germs, and destroying the usual flora necessary for vital functions, including those related to digestion and homeostasis in general [101];
2. Infection by *Clostridium difficile* [102];
3. Increased possibility of resistance to new antibiotics, which could be reserved for more special situations when carbapenems fail [103].

The problem is that in many scenarios, carbapenems already have a high percentage of resistance, higher than 20–25%. This is not generalizable, and there are geographical and local differences, but it is very common [93].

What is a fact in all epidemiological studies in ICUs is the increased influence of GNB MDR germs [77]. 

When it comes to deciding on an empirical therapy in this scenario of sepsis and septic shock, as we have already discussed, it is important to acknowledge that, although it is a *continuum*, they are two different entities. There is a reasonable consensus that, in the context of sepsis that occurs without shock, it is possible to wait a few hours to decide on the best treatment, to confirm that you have an infection, to obtain the relevant cultures, and to choose reasonable antibiotics which, of course, can also include carbapenems and new antibiotics [77]. The range of antibiotic selection in this case is wider and the need for breadth of coverage may be lower. The same guidelines of the SSC indicate the need to provide coverage against MRSA or coverage with two antipseudomonic drugs, although, in the absence of shock, it may be considered not to cover MRSA and to prescribe a single antipseudomonic [22].

However, all this changes in two scenarios:

1. In patients with shock and severe comorbidities;
2. In patients with significant risk factors for MDR germs, colonized by MDR, or admitted to a unit with a high percentage of resistance (the threshold of which is considered to be 20%).

In patients with shock and/or a context of high probability of infection by an MDR microorganism or colonized by an MDR, there is consensus on choosing an empirical treatment that covers, depending on the focus of suspicion, all possible germs that may have caused the condition, including those probable MDR germs, with the highest probability of success and administered in the most optimal possible doses and form [100]. 

For this reason, and depending on the context, the empirical antibiotic regimen should include in the treatment [104]

1. Coverage against MRSA, in general, and against enterococcus if it occurs in the context of intra-abdominal infection;
2. Hedging against GNB, especially difficult-to-treat *Pseudomonas* and MDR GNB, in general (ESBL producers and carbapenems, in particular), using at least two drugs during the first 48 h;
3. Assessing the need for an antifungal, especially in intra-abdominal infection with risk factors for invasive candidiasis.

## 10. Recommendations for Empiric Antibiotic Treatment of Patients in Septic Shock

### 10.1. Preliminary Considerations

**Figure 1:**
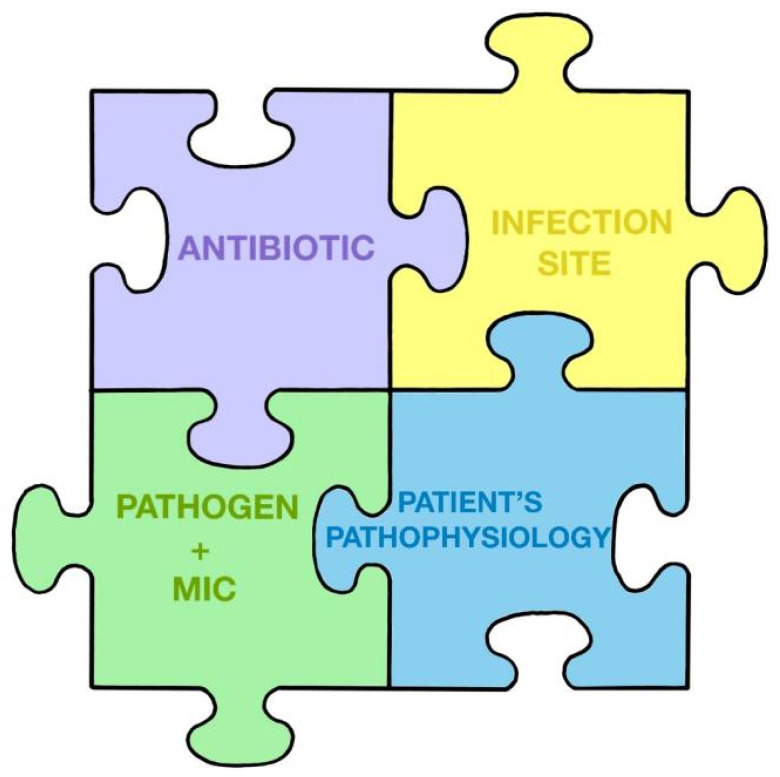
The puzzle of antibiotic treatment according to Pea and Viale.

When choosing the empirical guideline in each scenario, to try to personalize the treatment, we must consider the puzzle of Pea and Viale [105]. The antibiotic treatment of critically ill patients is like a children’s puzzle composed of several pieces that are combined to promote clinical cure and the prevention of the development of resistance: the antibiotic and/or antifungal and its properties, the site of infection, the pathogen responsible and its susceptibility to treatment (MIC), and the patient’s pathophysiology (Figure 1). 

The empirical guidelines will be greatly influenced by the suspicion of the focus of infection, since this can vary with, e.g., pneumonia or an intra-abdominal infection. 

In relation to the importance of reducing the bacterial load, it is interesting to know how the bacterial load affects the effect of antibiotics, recalling that when the bacterial load is very high, ceftazidime/avibactam may be the least affected in this context [106,107]. Finally, an important process, as Bassetti and Montravers point out, is to assess the local prevalence of MDR infections [96]. If the percentage of resistance to carbapenems is greater than 20%, an empiric antibiotic treatment strategy using new antibiotics would make sense, even in cases of sepsis and not only in septic shock. This should be applied, again, depending on the focus of infection. 

Therefore, decision making regarding the initial empiric antibiotic treatment of patients in septic shock should consider the following premises:

1. The bacterial load at the focus of infection will usually be high. The higher the bacterial load in the focus of infection, the higher the concentration of antibiotic needed to inhibit the growth of the microorganism and the greater the probability of selecting resistant mutants if the administered dose is not sufficient. The antibiotic exposure required to suppress the emergence of resistance should be maintained above the mutant selection window. Control of the outbreak via surgery or drainage is essential to decrease the bacterial load when possible.
2. As we do not know the microorganisms causing the infection or their patterns of antibiotic susceptibility, we can be guided by the suspected outbreak or guided by surveillance cultures, especially rectal exudate within the previous 48–72 h [99].
3. The local ecology of MDR bacteria influences the decision. A high percentage of resistance to carbapenems makes it necessary to consider the use of new antibiotics [77].
4. Empiric treatment should be active against all potentially involved microorganisms and, whenever possible, should contain a β-lactam antibiotic for its efficacy, spectrum, and bactericidal effect. Among the β-lactam antibiotics currently available, the most recommended for any outbreak, due to their antibacterial spectrum and probability of reaching the optimal target of Pk/Pd against MDR GNB, are ceftazidime/avibactam and meropenem. Depending on the focus of infection and colonization, personalization may be necessary and alternatives may exist: for example, in hospital-acquired pneumonia, we could include ceftolozane/tazobactam, and if the patient is colonized by metallo-beta-lactamase-producing germs or *Stenotrophomonases*, we could include cefiderocol [77].
5. The pharmacokinetics of antibiotic administration in sepsis will be drastically influenced by the inherent characteristics of the critically ill patient [108].
6. A patient in shock is at risk of irreversible damage, so it is urgent to reduce the bacterial load and control the immune response, while offering organ support.
7. Initial empirical therapy for patients in septic shock with pneumonia should be with two antibiotics and be based on risk factors for MDR pathogens discussed above, with an initial approach based on broad-spectrum therapy, followed by a reduction if MDR pathogens are ruled out in cultures [109].
8. Complicated intra-abdominal infection is usually polymicrobial, often with the intervention of GNB, anaerobes, and enterococci. Initial empiric therapy of patients in septic shock in this setting should include beta-lactam with a beta-lactamase inhibitor or carbapenem as well as coverage for anaerobes if beta-lactam does not cover them, coverage for enterococcus if the infection is nosocomial, and, in some patients with risk factors, additional initial coverage should be added for *Candida* species other than *C. albicans*. In this situation, it is key to control the outbreak, obtain quality samples, and process them quickly [110].
9. Daptomycin, linezolid, tedizolid, or vancomycin can be used against GPB. The choice depends on the location of the infection, renal function, and the need to use other nephrotoxic drugs simultaneously. It should be recalled that daptomycin does not show activity in the pulmonary focus [111].
10. Fungal infection accounts for 5% of sepsis. It is most commonly due to Candida spp. and can be predicted by specific scores such as the Candida Score as well as by epidemiological data, microbiological data, and biomarkers. Risk factors overlap with those of other causes of sepsis in the ICU. Preventive and empirical therapy is often necessary, and echinocandins are preferred for *Candida*, but some strains are becoming resistant so, in some scenarios, such as intra-abdominal infection or cases that are difficult to treat, liposomal amphotericin B may be a suitable alternative [112,113].
11. Appropriate cultures should always be obtained, depending on the focus of infection (blood cultures, rectal exudate, or other surveillance cultures, depending on the context), before the start or shortly after the initial empiric antibiotic treatment. The alarm, e.g., the sepsis code, should be triggered to ensure that samples are processed with rapid microbiological identification systems and, if possible, the microbiologist on duty should be alerted and interacted with [28].
12. After the first 24–48 h, the treatment should be re-evaluated in the context of a decrease in bacterial load and clinical improvement. Rapid diagnostic techniques will have allowed us to assess the absence or presence of ESBL-producing enterobacteriaceae or carbapenemases, resistant *P. aeruginosa*, MRSA, as well as a low value of β-D-glucan or its positivity, which will allow us to narrow the antibiotic spectrum or suggest a need to change treatment [114].
13. Short-term treatments should be attempted. Biomarkers such as procalcitonin (PCT) can help us in the application of short antibiotic treatment regimens [115].

### 10.2. Individualization of Antibiotic Treatment in Critically Ill Patients

Although the main challenges, in this context of the high prevalence of resistant strains, are to avoid delaying diagnosis through the rapid identification of microorganisms and their sensitivity patterns, adequate treatment is complicated by the peculiarities of the most severely ill patients who present alterations in their extracellular volume, renal function, and immunity, among other symptoms. In this way, the severity of their condition not only produces a higher mortality rate per se, but also affects the clinical response to antimicrobial treatment. If we add to this the controversies regarding the available evidence in other aspects of antimicrobial treatment, such as the use of combination therapies, the duration of treatment, the difficulty of de-escalation, and the existing means in each workplace with respect to clinical, laboratory, and organizational tools, there will undoubtedly not be one size that fits all, and a good place to start will be personalizing the treatment to the clinical circumstances and microbiological data of each patient and each center [116]. 

What would it consist of, then, to individualize antibiotic treatment in critically ill patients in order to achieve maximum effectiveness?

“Optimization” is looking for the best way to perform an activity or improve the performance of something. 

“Effectiveness” is defined as the ability to achieve the desired or expected effect. 

To “individualize” is to attribute to someone or something characteristics that differentiate them from others. 

**Figure 2:**
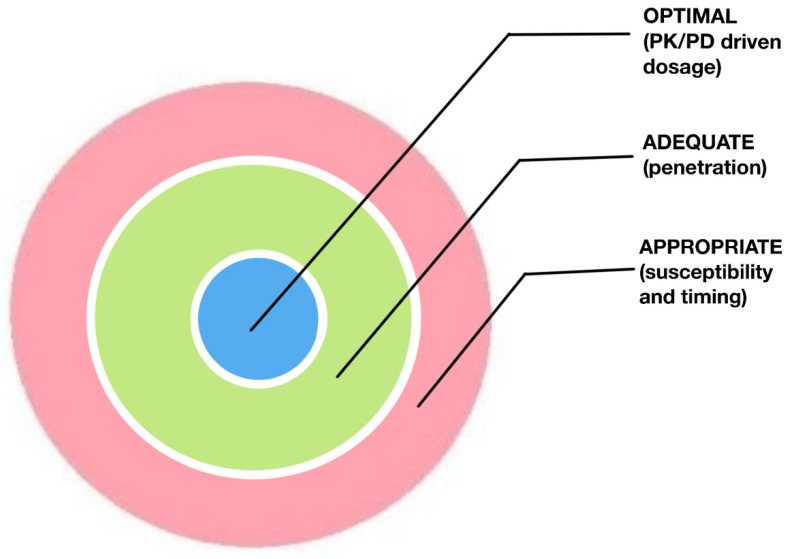
Appropriate/adequate/optimal antibiotic treatment.

As our main initial goal must be the rapid elimination of the pathogen, achieving the maximum efficacy of antibiotic treatment in each patient becomes our priority. Although the literature often speaks in terms of antibiotic coverage, there is emerging evidence to suggest that the in vitro sensitivity of the microorganism to the antibiotic is critical and that knowing this sensitivity is not enough to achieve the best results. Therefore, fixed regimens, independent of the site of infection and the pathophysiological conditions of the patients, may not obtain the expected results and may be ineffective. To this end, we will distinguish, as proposed by different authors, these different levels of success in Figure 2 [117,118].

(a) Appropriate: defined by in vitro susceptibility to the causative pathogen and early administration of anti-infective treatment. That which is in line with published consensus and guidelines is also considered appropriate.
(b) Adequate: in addition to the “appropriate” characteristics, the level of success is considered adequate if pharmacokinetic and pharmacodynamic strategies are applied and taken into account, such as shorter administration intervals, assessment of physicochemical characteristics in the approach to the different sites of infection, monitoring of levels, avoiding interactions, etc.
(c) Optimal: in addition to the “appropriate and adequate” characteristics, this level of success consists of administering a dose that allows penetration into the site of infection at an effective concentration for the eradication of the infection.

In critically ill patients, the probability of obtaining insufficient concentrations of the drug for microorganisms that are sensitive in vitro, but with an MIC very close to the cut-off limit, sometimes causes no efficacy to be observed in vivo, thus leading to therapeutic failure and the emergence of resistance. It is difficult to achieve optimal antibiotic treatment regimens in critically ill patients without significantly changing dosage [39,105].

### 10.3. Early Antibiotic Treatment in Patients in Septic Shock

The current standard of antibiotic treatment for critically ill patients is based on early treatment and involves the administration of antibiotics in less than an hour in the most severely ill patients or those presenting with septic shock [22].

Although most of the available data are not extracted from clinical trials, due to the obvious ethical difficulties in being able to randomize the delay of antibiotic treatment, since the publication in 2006 of the classic retrospective observational study (in which Kumar showed that among 2154 patients with septic shock, for every hour of delay in antimicrobial therapy, there was an average decrease of 7.6% in survival), all guidelines have strongly recommended this strategy of precocity [119].

Subsequent major observational studies have confirmed these results. One of them, a multicenter and international study with data from 17,990 patients with septic shock receiving antibiotics, confirmed that delay in the administration of antibiotics was associated with higher in-hospital mortality [120]. There was a linear increase in the risk of mortality for each hour of delay in antibiotic administration from the first to the sixth hour after patient identification in this study.

Recently, using the new definitions of sepsis, a study of 3035 patients demonstrated that for patients with septic shock, the administration of broad-spectrum antibiotics within 1 h of sepsis recognition reduced in-hospital mortality. However, in patients with sepsis without shock, the association between antibiotic administration within 1 h and in-hospital mortality was not statistically significant. In linear regression models limited to patients who received antibiotics within the first 3 h, patients with septic shock showed an increased risk of mortality for each hour of delay in antibiotic administration, but no such trend was observed in those without shock [121].

It is true that doctors do not intentionally delay the administration of broad-spectrum antibiotic treatment, but it is also true that there are difficulties in being able to make a rapid diagnosis and that it can be difficult for us to recognize all high-risk patients. It is often simpler for doctors to administer antibiotics earlier to those cases that are more severe, with more comorbidities and clearer symptoms of infection [122]. In addition, there may be operating habits in hospitals that can act as barriers to early antibiotic administration [123]. 

Barriers to early antibiotic administration:

- Lack of training;
- Lack of correct assessment of severity and delay in recognition of sepsis;
- Increased work;
- Transfer of the patient (to the operating room, to tests, etc.);
- Possibility of atypical manifestations, without fever or confusion;
- Delays due to other diagnostic tests and slow collection of microbiological samples;
- MDR germ infections and not recognizing MDR risk factors;
- Waiting to carry out blood cultures before providing the antibiotic;
- Very importantly, failing to recognize that providing an inappropriate antibiotic is like giving none or even worse. Appropriate antibiotics should not be delayed by inappropriate administration;
- Not specifying the order in which antibiotics are administered and what the broad-spectrum “key” antibiotic is;
- Needing to obtain authorization for the prescription, either from another specialist, the head of the infectious diseases department, or the pharmacy, before starting treatment.

Approach to avoid delays in antibiotic administration:

- The presence of hypotension in a patient with suspected infection should be considered septic shock in the absence of an alternative explanation;
- Failure to transfer or transport the patient before antibiotic treatment has been administered;
- If an antibiotic is requested in these situations, it must be ensured that it is administered immediately (it has been reported that the delay from the time the treatment is written to the time it is administered can be up to 3 h on average);
- Combination regimens should be administered with the key antibiotic (the broadest spectrum) provided first;
- Promote education, training, and teamwork to reduce administration time.

Early antimicrobial therapy can have a negative impact in terms of toxicity and costs, especially in less severe infections and sepsis without shock, and can contribute to a delay in diagnosis in some initially uninfected patients, such as critically ill surgical patients, in whom it is particularly difficult to distinguish between the effects of inflammatory response due to surgery or other pathological circumstances and that due to infection. 

Recently, more value has also been granted to the impact of antibiotic treatment in the ICU on the microbiota, to the influence of dysbiosis on immunity and patient outcome, and to strategies to maintain regular gut flora, but this is beyond the scope of this review.

The reality is that the literature on the association between time to antibiotic treatment and mortality in sepsis is controversial, almost exclusively observational, and at high risk of bias. Sources of bias include a lack of differentiation between sepsis and septic shock, insufficient adjustment for potential confounders, and the combination of the high increases in mortality associated with very long delays until disease occurs [124]. 

Recent meta-analyses have not been conclusive about the benefit of antibiotic treatment, so strictly from the point of view of scientific evidence, it cannot be recommended that antibiotic administration be extremely rapid, at least in sepsis; however, perhaps this is not the case in septic shock [125]. Nevertheless, the fact that the antibiotic should be administered in the first few hours is still mandatory in the recommendations of the SSC, although it has led to criticism by the Infectious Diseases Society of America (IDSA) regarding this precocity in patients without shock [126]. In the future, it may be necessary to clarify what the true definition of “early” is, what the time window is in which the administration of antibiotics is most effective in controlling the burden of infection, and how to interpret the spectrum of severity, extreme urgency, and speed required for administration. There appears to be a reasonably strong relationship between hourly antibiotics and septic shock mortality but a less pronounced relationship for sepsis without shock. There are pieces of data demonstrating that intervals of up to 90 min for antibiotic administration in patients with sepsis without shock do not make a difference. It appears that the risk of increased mortality in patients with non-shock sepsis increases at approximately 3–5 h until antibiotics and thereafter, but data differentiating the precise impact from 3 to 5 h intervals are few and imperfect. In the case of septic shock, although there are numerous biases in most studies, and the linear relationship between delay and mortality is clearly a statistical association and a product, among other things, of not using confounding factors in the analysis, it does seem that precocity is more important [127]. 

With what has been said so far, perhaps a good place to start, at the present time, would be continuing to administer antibiotics rapidly, for example, always within the first 3 h, as suggested by the IDSA in its criticism of the recommendation of administration in the first hour of EFS. However, perhaps this should be carried out while attempting to acquire a rapid diagnosis prior to administration (or, at least, to obtain early and profitable samples) and to have sufficient time to reflect and consult, if necessary, on the best antimicrobial regimen for each patient. In the most severe cases of septic shock, it is reasonable to expend the maximum effort so that the optimal time for administration occurs before the third hour and closer to the first hour since the rapid decrease in the bacterial load through control of the focus of infection and since appropriate and early antibiotic treatment is key.

### 10.4. The Importance of Focus Control in Surgical Units

The management of infection in the ICUs of surgical patients presents its own challenges since, as in other settings, diagnosis is often problematic and multidrug resistance and pharmacokinetic changes present challenges. The need to control the focus of infection, either by surgical and/or percutaneous procedure, adds complexity to the case [128]. 

Restoring anatomy and function is equally important and can be carried out in the same operation, although sometimes the decision to perform it in this way is difficult. As a rule of thumb, measures to control the focus of infection should not be delayed except in those situations where demarcation of non-viable tissue is preferable, such as in infected necrotic pancreatitis or in situations where the source of control is difficult to access. Nowadays, there is a renewed interest in so-called damage control surgery in intra-abdominal infection, which involves the initial control of the infection through an abbreviated laparotomy and the use of an initial technique that leaves the laparotomy open in a way that minimizes the risk of damage, facilitates the cleaning of the peritoneal cavity, and decreases the risk of abdominal hyperpressure and compartment syndrome [129]. The definitive anatomical reconstruction will be performed later, at 24–72 h. In this approach, the choice of the temporary abdominal closure system seems to be important, with negative pressure therapy being a common option as it produces fewer fistulas, fewer abscesses, and a higher survival rate [129].

The SSC guidelines recommend identifying an anatomical source of infection that may require control of the outbreak and resolving it as soon as possible from a logistical and medical point of view. The approach should be individualized: imaging tests are very important and should not be limited to the abdominal cavity [130]. There is no point in delaying such control unless there are significant metabolic or coagulation alterations or the patient is very unstable hemodynamically. The appropriate timing to control the outbreak is a matter of debate, and one should adopt a multidisciplinary approach guided by the severity of the infection, the speed of deterioration of the patient, the presumed source of infection, and the pathophysiological state of the patient. There are pieces of data indicating that in the most severe infections, controlling the focus of infection produces better results when it is carried out between 2 and 6 h after diagnosis [131]. Again, the stewardship concept, although applied on this occasion to the creation of teams dedicated to the control of the focus with the availability of diagnostic and therapeutic methods, including interventional radiology, 24 h a day, can help to select more appropriate treatments and implement surgical strategies that improve outcomes in these patients [132].

Surgical infections, specifically abdominal infections, are the most complex among nosocomial infections: they involve longer average stays, more patients in shock, and a greater occurrence of renal failure with higher mortality. Their microbiological pattern is usually polymicrobial with a non-negligible presence of fungi, difficulties in establishing the pathogenic role of each of them and their contribution to the patient’s overall condition, and a growing presence of multidrug-resistant organisms, especially carbapenemase-producing organisms [110]. Inflammation associated with surgical trauma means that the traditional criteria of systemic inflammatory response are present on a regular basis and are, therefore, of little use in the diagnosis of an infectious process, as are the analytical markers of infection. Similarly, there is little use in measurements regarding leukocytosis, PCT value (although an increase in the latter, doubling its initial value in the first 24 h after surgery, can help to warn of a failure to control the focus of infection), or other signs, such as hypotension or oliguria, which may be related to postoperative complications of non-infectious origin [133]. All of this complicates the decision-making process regarding whether or not to start antibiotic treatment and with which drugs. In addition, if the patient’s serious situation is prolonged, de-escalation will also be difficult. If excellent control is assumed, antibiotic treatment of abdominal infection should be limited to 3–5 days. This seems to be demonstrated in nosocomial infections, even in patients with APACHE > 10, with no increase in relaparotomies or increases in mortality [134]. Therefore, clinical suspicion continues to appear as the best weapon to monitor a possible failure in the control of the focus of infection. Adding organ dysfunction parameters (SOFA score) could identify patients who will require additional control of the focus of infection. Imaging techniques such as ultrasound and CT are also very important here since they add the possibility of percutaneous drainage of collections susceptible to organ dysfunction if qualified personnel are available on a continuous basis [135]. 

Quantifying residual infection after controlling the focus of infection can help assess the role certain interventions play and guide antibiotic therapy. For these purposes, residual infection generally refers to the incomplete percutaneous drainage of an abscess or residual necrotic material that cannot be debrided, and ongoing contamination refers to the source of the infection (e.g., perforation that cannot be transformed into a fistula and continues to drain into the abdominal cavity) [136].

### 10.5. Optimization of Antibiotic Treatment According to Kumar’s Hypothesis

Due to their relevance in the current conception of sepsis and their possible implications in the choice and administration of antibiotic therapy, we will dedicate a section to commenting on Kumar’s theories [106].

After witnessing a decrease in mortality rates from infectious causes with the advent of penicillin, the absolute mortality figures remain constant today despite the emergence of new antimicrobials. This is due to a higher prevalence of resistant microorganisms, a higher incidence of sepsis in older populations, and the existence of more aggressive microorganisms [137].

Other supportive measures and treatments unrelated to antibiotics have been questioned following dozens of studies. Kumar’s hypothesis is that tested therapies have failed because the disease process has not been properly interpreted [138]. The alternative pathophysiological model proposed by Kumar suggests the substantial implication of bacterial load as the main driver of organ dysfunction and proposes that bacterial load should, therefore, guide the antibiotic regimen in sepsis. The key to improving outcomes may then be to reduce this burden as soon as possible based on better administration of available antibiotics and other strategies that aim at this end. Therefore, the early administration and use of bactericides becomes fundamental in this model.

The microbiological model of sepsis as well as the immunological model are not able to fully explain the pathophysiology of the disease. This is understandable when we consider a key element in the mortality of septic states, the concept of irreversible shock, which suggests that shock, regardless of its etiology, can only be tolerated for a limited time and that, once presented, it becomes irreversible, with inevitable progression to chronic critical illness or death if not reversed in a short period of time. A parallel concept in resuscitation is hemodynamic incoherence.

This concept is related to the golden hour demonstrated in traumatic hemorrhagic shock but is applicable to various forms of critical injury. 

A conceptual model that incorporates the key elements of this sepsis model must consider the following:

- First, the understanding that there is a threshold at which, when reached, inflammatory mediators will be associated with cellular dysfunction and tissue damage manifested as septic shock and that this threshold may be variable among individuals: those with poor cardiopulmonary reserve will go into shock earlier and at lower levels of cellular dysfunction or tissue damage while young, healthy patients may require a substantially higher degree of inflammation to reach the same shock threshold.
- Second, that the appearance of a new element, such as the presence of shock commonly manifested as recurrent or persistent hypotension—especially if it manifests itself with poor perfusion that does not recover and hemodynamic incoherence—in turn, places patients on the path to irreversible organ damage. At some indeterminate point after the onset of hypotension, depending on the degree of hypotension, the contributions of its comorbidity, and the patient’s genotype, the patient will be at high risk of chronic critical illness or death.

Due to genetic variations in the host and pathogen and the clinical variability of the infection, the exact point at which sepsis becomes irreversible for each patient cannot be known in advance today; yet, the progression will be similar for all patients who reach it. 

**Figure 3:**
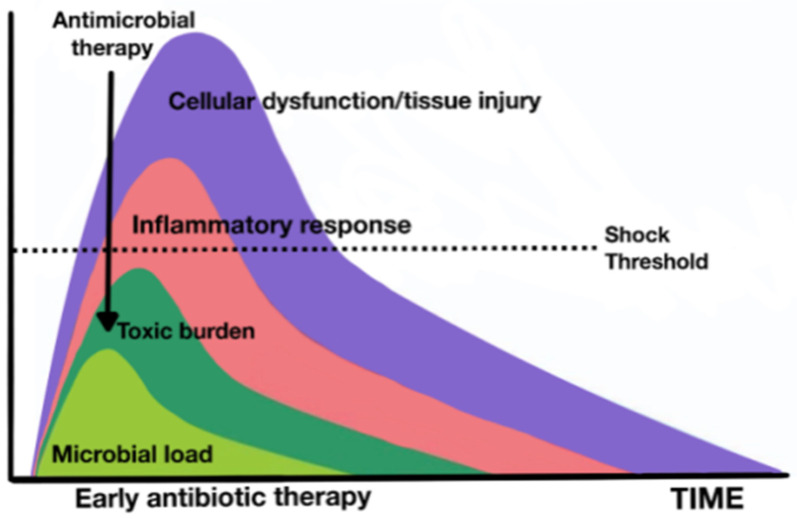
Pathophysiological model of Kumar’s sepsis: early antibiotic therapy.

**Figure 4:**
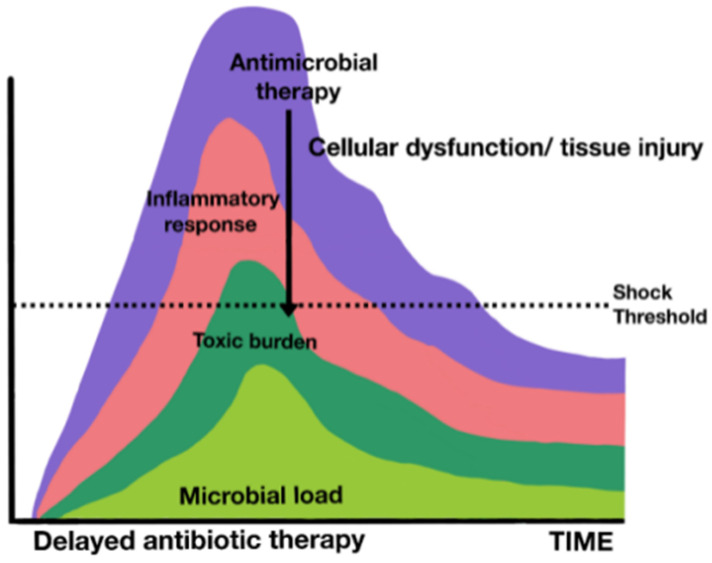
Pathophysiological model of Kumar’s sepsis: delayed antibiotic therapy.

Following Kumar’s model, the underlying focus in septic shock is the total microbial load. The speed of achieving its reduction to a subcritical threshold after the onset of infection will be of paramount importance in survival, thus minimizing the period in which there are enough microorganisms to generate shock, limiting the risk of reaching a point where recovery is no longer possible (Figure 3 and Figure 4).

This model suggests that septic shock and sepsis are two different entities rather than a continuum of the same disease. The main difference between them lies in the time available until the onset of irreversible and irreplaceable organ failure. The simple evidence of observation, in both cases, of the clinical behaviors of hypotension, lactic acidosis, and fatigue of compensatory responses, added to the large differences in mortality rate between septic shock (greater than 40%) and sepsis (around 10%) invite us to think about these as two conditions, according to Kumar, as different entities.

Thus, and according to this model, the relevant factors to consider for the clearance of pathogens in septic shock are

1. Early antimicrobial therapy:
  - Start antimicrobial therapy early;
  - Use a loading dose.
2. Antimicrobial potency:
  - Ensure bactericidal antimicrobial activity;
  - Optimize pharmacokinetic indices;
    - Time-dependent agents;
    - Concentration-dependent agents;
  - Use combination therapy with antibiotics that have different mechanisms of action.
3. Supplementary therapies: focus control and resuscitation to ensure perfusion and hemodynamic coherence.

### 10.6. Duration of Antibiotic Treatment within Stewardship Programs

Within stewardship programs, there are several reasons to try to shorten the duration of antibiotic treatment. Alterations in the microbiota increase with each day of antibiotic treatment. The consequences of this alteration are not well studied, but it could contribute to an increased risk of sepsis in the following months [139]. The increased incidence of adverse events is also a cause for concern, including the emergence of antimicrobial resistance, acute renal failure, or *C. difficile* infections. Each day that antibiotic treatment is prolonged, the risk increases [140].

Regarding the early suspension of antibiotic treatment, we must take into account two different situations. On the one hand, in patients with suspected infection but in whom cultures extracted before the start of antibiotic treatment are negative or demonstrate non-significant isolation, antibiotic treatment should be discontinued. In patients with suspected ventilator-associated pneumonia but with negative bronchoalveolar lavage (BAL) cultures, Raman et al. suggested early discontinuation of antibiotic treatment, finding no difference in mortality (4 days of treatment vs. 9 days). Meanwhile, the appearance of multidrug-resistant bacteria was clearly less frequent in the short-term treatment group (7.5% vs. 35.7%) [141]. In critically ill patients with microbiological confirmation of infection, there is evidence demonstrating the non-inferiority of short treatment regimens over long treatment, mainly in nosocomial pneumonia and associated with mechanical ventilation and postoperative intra-abdominal infection, with 7 days of antibiotic treatment being sufficient in most cases [134]. A clinical trial carried out with patients with complicated intra-abdominal infection (they were not exclusively critically ill patients) even showed that the duration of antibiotic treatment could be safely reduced to 3–5 days, provided that adequate control of the focus of infection was ensured [142]. In a study conducted in hemodynamically stable patients with GNB bacteremia, Yahav et al. demonstrated non-inferiority of a short treatment regimen of 8 days versus a long regimen of 14 days [143]. In bacteremia not complicated by *S. aureus*, current recommendations continue to indicate the need for 14 days of antibiotic treatment, but there is currently a clinical trial underway comparing a duration of 7 days versus 14 days [144], and there are observational studies that support the short regimen in patients with S. aureus bacteremia in the absence of endocarditis, distant metastases, or the presence of prostheses [145]. 

Chastre et al. found no difference in mortality in short-term treatment in patients with ventilator-associated pneumonia or in the rate of infection recurrence except in pneumonia caused by *P. aeruginosa,* which was higher in the short-term treatment group [146]. Another recent clinical trial, which was halted due to lack of patient recruitment, compared treatment durations of 8 days versus 15 days in patients with ventilator-associated pneumonia caused by *P. aeruginosa*. These researchers could not demonstrate the non-inferiority of the short treatment regimen in terms of the primary outcome of lung infection recurrence and mortality [147]. Despite these results, an optimal duration of antibiotic treatment of 7 days could be established, even in *P. aeruginosa* infections, as the potential benefit of prolonging antibiotic treatment is probably outweighed by the harms caused by unnecessary prolongation of antibiotic treatment [148]. Patients admitted to the CCU due to COVID-19 should probably make individualized decisions, and it may be necessary to prolong antibiotic treatment in certain cases due to the very high rate of secondary infections and recurrence [149].

The use of biomarkers can also help to shorten the duration of antibiotic treatment in critically ill patients. PCT is a biomarker that increases mostly during bacterial infections but also, to a lesser degree, in inflammatory response or surgical trauma [150,151]. Several clinical trials have shown that the duration of PCT-adjusted antibiotic treatment can be safely reduced in critically ill patients. The trials that included the most patients were those conducted by Bouadma et al. and De Jong et al. Bouadma et al. demonstrated that patients had more antibiotic-free days in the PCT group, in which antibiotic treatment was discontinued when PCT levels were less than 0.5 ng/mL or had been reduced by at least 80% of the peak concentration [152]. De Jong et al. demonstrated, in 1575 critically ill patients, that the use of the same protocol was able to reduce the mean duration of antibiotic treatment to 5 days compared to 7 days in the control group and even found a reduction in mortality at 28 days [153]. An observational study showed that in critically ill post-surgical patients with secondary peritonitis, the PCT-based algorithm could be useful to shorten the duration of antibiotic treatment (5.1 days vs. 10.2) [133].

It has recently been suggested that calprotectin may be superior to procalcitonin as a biomarker in sepsis and as an indicator of the need for intensive care in these patients [154,155].

Our general recommendation for the duration of antibiotic treatment in critically ill patients is 7 days, which could be further reduced by directing treatment with PCT. In the case of intra-abdominal infection, with adequate control of the focus of infection, the duration could be 5–7 days [156].

### 10.7. Tools for Making Sound Decisions

Among the tools available to improve the care of critically ill, infected patients are cognitive aids, the paradigmatic example of which are published antibiotic treatment guidelines. We will highlight two of these: the Mensa guide and the Sanford guide. 

The Antimicrobial Therapeutics Guide (Mensa) (Mensa Guide—Apps on Google Play) is the most widely used guide in Spain, with more than 20 editions. It is very intuitive, complete, and easy to use. Among the advantages we found, the following stand out, among others:

- Annual update;
- Provides local epidemiology data (in Spain, with our frequent germs and our resistances);
- Characteristics of antimicrobials;
- Dosing of antimicrobials in special situations;
- Treatment of infections caused by specific microorganisms.

The guide is available in an electronic app that even provides dose adjustment based on pharmacokinetic, pharmacodynamic, and MIC parameters. It is only available in Spanish.

The Sanford Guide to Antimicrobial Therapy (Sanford) (Apps on Google Play) is like the “Mensa Guide” in aspects such as the annual update (more than 40 editions) and its availability in a marketed app, but with notable differences:

- It is a guide made in the USA, available in English;
- Epidemiology contains data from the United States.

The biggest difference is in searching for information as the Sanford guide uses tables.

## 11. Executive Summary for the Individualization of Antibiotic Treatment in Critically Ill Patients

Different studies confirm the relationship between delay in initiating appropriate antibiotic treatment and mortality [97,119]. In GNB infections, inappropriate antibiotic treatment increased the risk of mortality by almost four times [157]. In addition, the need to quickly determine correct antibiotic treatment is extremely important in patients with sepsis or septic shock for whom, even with adequate antibiotic treatment, mortality can reach between 27% and 40% in patients with multiple comorbidities and a functional reserve limited by their frailty and in patients with some degree of immunosuppression [158]. Despite the importance of these data, the reality is that the rate of inappropriate antibiotic treatment remains, according to Vázquez-Guillamet et al., at almost 30% of patients with sepsis or septic shock, and, according to these authors, the number of patients required for appropriate antimicrobial treatment to save a life would be five [159]. The most important factor that predisposes a patient to inappropriate antibiotic treatment is infection with MDR microorganisms [157].

Knowledge of local epidemiology is essential to initiate appropriate empirical treatment. Knowing the total rate of carbapenem resistance of GNB with epidemiological importance in each service and hospital can be used as an indicator of risk for the patient of the presence of colonization or infection by these microorganisms. As previously discussed, a threshold of 10–20% resistance to carbapenems is considered sufficient to initiate active antimicrobial treatment with new antimicrobials like sparingly used carbapenems. They can also be used if the incidence of resistance is less than 10–20% because they are very good antibiotics per se [96]. 

Most hospital-acquired infections are infections that originate from the endogenous microbiota of the mucosal surfaces via translocation or invasion of the predominant microorganisms, depending on the density of the bacterial population. Therefore, knowing the colonizing flora and their pattern of antimicrobial susceptibility may be important in the choice of initial empirical treatment. For all these reasons, it would seem reasonable to perform surveillance cultures upon admission to the ICU and subsequently one to two times a week, although changes in the composition of the microbiota before the sepsis episode occurs cannot be ruled out. An alternative strategy is to obtain a semi-quantitative rectal, pharyngeal, and nasal mucosal smear at the time of sepsis.

It is also important to assess the site of infection. In patients with risk factors for carbapenem-resistant GNB, the use of new antibiotics should be considered when the clinical efficacy of possible alternatives is expected to be suboptimal, as is the case with polymyxins and/or aminoglycosides in patients with pneumonia [160,161].

However, decision making about the use of active empirical treatment versus CRE can be difficult for the clinician. Scales have been published and validated to predict the individual risk of developing bacteremia in patients colonized by these microorganisms [162,163,164,165]. These scales have their limitations, in the sense that they are validated in an epidemiological setting with a specific group of patients and they cannot necessarily be reproduced in different clinical situations, leading to a high negative predictive value but a limited positive predictive value.

In any case, it is crucial to initiate early empirical antibiotic treatment in which there is no margin for error in those patients with nosocomial infection and septic shock, in patients with sepsis and a low functional reserve due to their frailty, or in immunosuppressed patients. For these types of patients and for those in whom—due to the local ecology, individual history of colonization, previous infection, and risk factors for CRE such as the presence of multiple comorbidities, the best option would be to initiate an active empirical treatment against CRE (within 24–36 h and using the information obtained from the cultures) [166]—a definitive empiric or targeted treatment could be decided, unnecessary overuse of these antibiotics could be avoided.

**Figure 5:**
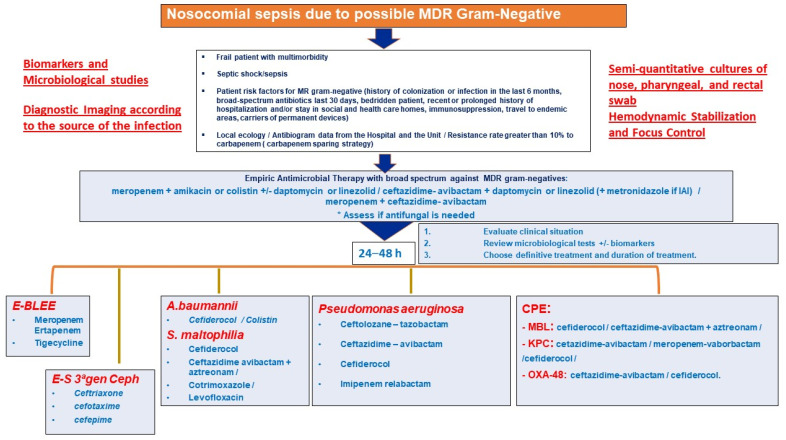
Proposal for antibiotic treatment of nosocomial sepsis that is likely due to resistant Gram-negative microorganisms.

We need antibiotics that are active against as many GNB as possible implicated in carbapenem resistance. Depending on the focus of infection, we should add antimicrobials with activity against GPB (daptomycin, linezolid, and vancomycin) and anaerobes, as in the case of intra-abdominal infection (tigecycline or metronidazole) [167]. Figure 5 graphically summarizes the possible factors that determine the choice of new antibiotics in empirical and focused antimicrobial treatment against carbapenem-resistant GNB in nosocomial infection.

Bearing in mind that, until very recently, we did not have antimicrobial options against this type of infection that were completely effective and well tolerated, it is necessary to optimize the use of new antibiotics by guaranteeing the best available treatment to patients while delaying the emergence and spread of resistance.

## 12. Conclusions

The challenges in antibiotic treatment in sepsis include adequate stewardship programs based on knowledge related to the infection in intensive care and in the context of multi-resistant germs, especially Gram-negative germs, based on sparing carbapenems and the empirical and rational use of new antibiotics.

## Figures and Tables

**Table 1:** The new antibiotics: dosage, indication, and spectrum.

Drug	Usual Dosing Regimen in Sepsis	Indication	Activity > 80%
Ceftaroline	600 mg q8 h IV	CAP, cSSTI	MRSA
Ceftobiprole	500 mg q8 h IV	HAP, CAP	MRSA
Ceftazidime/avibactam ^1^	2 g/0.5 g q8 h IV	BSI, HAP, VAP, cIAI, UTI	ESBL, Amp C, KPC
Ceftolozane/tazobactam ^2^	1.5 g q8 h/3 g q8 h (VAP) IV	BSI, UTI, cIAI, HAP, VAP	ESBL, Amp C
Aztreonam/avibactam ^3^ (ATM/AVI)	6500 mg ATM/2000 mg AVI q24 h on day 1 followed by 6000 mg ATM/2000 mg AVI q24 h IV	HAP, VAP, BSI, UTI, cSSTI, cIAI, BSI	ESBL, Amp C, KPC, Metallo-β, *Stenotrophomonas*
Meropenem/vaborbactam	2 g/2 g q8 h IV	UTI, cIAI, HAP, VAP	ESBL, Amp C, KPC
Cefiderocol	2 g q8 h IV	BSI, HAP, VAP, cIAI, UTI	ESBL, Amp C, KPC, Metallo-β, OXA-48, *Pseudomonas*, *Stenotrophomonas*
Imipenem/relebactam ^4^	500 mg/250 mg q6 h IV	BSI, HAP, VAP, cIAI, UTI	ESBL, Amp C, KPC, *Pseudomonas*
Eravacycline	1 mg/kg q12 h IV	cIAI	ESBL, Amp C, KPC, Metallo-β, OXA-48, *Acinetobacter*, *Stenotrophomonas*
Plazomicin ^5^	15 mg/kg q24 h IV	In combination for BSI, UTI, HAP, VAP	ESBL, Amp C, KPC, OXA-48
Tedizolid	200 mg q24 h IV, oral	cSSTI, HAP	MRSA
Cefepime/taniborbactam ^6^	2 g/0.5 g q8 h IV	UTI, HAP, VAP	ESBL, Amp C, KPC, OXA-48, *Stenotrophomonas*
Cefepime/enmetazobactam	2 g/0.5 g (phase 3 studies)	UTI; phase 3 studies	ESBL, Amp C, KPC, OXA-48
Cefepime/zidebactam ^7^	2 g/0.5 g (phase 3 studies)	phase 3 studies	ESBL, Amp C, KPC, OXA-48, *Acinetobacter*, *Stenotrophomonas*
Temocilin	2 g/8 h IV (phase 3 studies)	phase 3 studies	ESBL, Amp C, KPC

CAP: community-acquired pneumonia; cSSTI: complicated skin and soft tissue infections; BSI: bloodstream infection; HAP: hospital-acquired pneumonia; VAP: ventilator-associated pneumonia; cIAI: complicated intra-abdominal infection; and UTI: urinary tract infection. MRSA: methicillin-resistant *Staphylococcus aureus*; ESBL: extended-spectrum beta-lactamase; KPC: Klebsiella pneumoniae carbapenemase; activity 30–80%: Metallo-β: 5, 6, 7; OXA-48: 3, 4; *Pseudomonas*: 1, 2, 3, 5, 7; *Acinetobacter*: 7; and *Stenotrophomonas*: 2.

**Table 2:** Antimicrobial activity spectrum chart.

	ESBL Producer	Amp C Producer	KPC-Type Producer	NDM-Type Producer	OXA-48-like Producer	Carbapenem-Resistant *P. aeruginosa*	Carbapenem-Resistant *A. baumannii*	*S. maltophilia*
Colistin/polymixyn								
Fosfomycin								
Tigecycline								
Ceftazidime/avibactam						(1)		
Ceftolozane/tazobactam						(1)		(1)
Imipenem/relebactam								
Meropenem/vaborbactam					(1)	(1)		
Cefepime/taniborbactam								
Cefepime/enmetazobactam								
Cefepime/zidebactam								
Aztreonam/avibactam								
Cefiderocol								
Eravacycline								
Plazomicin								
Temocilin								
Ampiciline-sulbactam								

Color code: green, activity > 80%; orange, activity 30–80%; red, activity < 30%; and white, not evaluated/no data found. ESBLs: extended-spectrum beta-lactamases; NDM: metallo-beta-lactamase New Delhi type; KPC: *Klebsiella pneumoniae* carbapenemase; OXA-48-like: oxacillinases with D carbapenemase activity; and (1) the percentage of activity of these antibiotics in these situations may vary slightly in one direction or another depending on the series considered and the prevalence of resistant individuals in certain geographic regions.

## Abbreviations

WHO	World Health Organization
GAP-AMR	Global Action Plan for the Management of Antibiotic Resistance
ICUs	Intensive care units
AMS	Antimicrobial Stewardship
SSC	Surviving Sepsis Campaign
UK	United Kingdom
POC	Point-of-care
MIC	Minimum inhibitory concentration
EUCAST	European Committee on Antimicrobial Susceptibility Testing
BP	Breakpoint
MPC	Mutant preventive concentration
Pk/Pd	Pharmacokinetic/pharmacodynamic
MDR	Multidrug resistance
RCT	Randomized controlled trial
PI vs. SI	Prolonged infusion (PI) vs. short infusion (SI)
ATS	American Thoracic Society
CDC	Centers for Disease Control and Prevention
ESCMID	European Society of Clinical and Infectious Diseases
BL-BLI	Beta-lactams with beta-lactamase inhibitor
TDM	Therapeutic drug monitoring
ESBL	Extended-spectrum beta-lactamase
UTI	Urinary tract infection
GPB	Gram-positive bacteria
GNB	Gram-negative bacteria
TMP-SMX	Trimethoprim-sulfamethoxazole
FDA	Food and Drug Administration
EMA	European Medicines Agency
CRE	Carbapenem-resistant Enterobacterales
MRSA	Methicillin-resistant *Staphylococcus aureus*
IDSA	Infectious Diseases Society of America
PCT	Procalcitonin

## References

[1] Cortegiani A., Antonelli M., Falcone M., Giarratano A., Girardis M., Leone M., Pea F., Stefani S., Vaggi B., Viale P. (2023). Rationale and clinical application of antimicrobial stewardship principles in the intensive care unit: A multidisciplinary statement. J. Anesth. Analg. Crit. Care.

[2] Liebchen U., Briegel J., Brinkmann A., Frey O., Wicha S.G. (2023). Individualised dosing of antibiotics in ICU patients: Timing, target and model selection matter. Intensive Care Med..

[3] Yoo J.H. (2018). The Infinity War: How to Cope with Carbapenem-resistant Enterobacteriaceae. J. Korean Med. Sci..

[4] Tang K.W.K., Millar B.C., Moore J.E. (2023). Antimicrobial Resistance (AMR). Br. J. Biomed. Sci..

[5] Llor C., Bjerrum L. (2014). Antimicrobial resistance: Risk associated with antibiotic overuse and initiatives to reduce the problem. Ther. Adv. Drug Saf..

[6] Diekema D.J., Hsueh P.R., Mendes R.E., Pfaller M.A., Rolston K.V., Sader H.S., Jones R.N. (2019). The Microbiology of Bloodstream Infection: 20-Year Trends from the SENTRY Antimicrobial Surveillance Program. Antimicrob. Agents Chemother..

[7] Leone M., Duclos G., Lakbar I., Martin-Loeches I., Einav S. (2023). Antimicrobial resistance and outcome in the critically ill patient: An opinion paper. J. Crit. Care.

[8] Karukappadath R.M., Sirbu D., Zaky A. (2023). Drug-resistant bacteria in the critically ill: Patterns and mechanisms of resistance and potential remedies. Front. Antibiot..

[9] Paterson D.L. (2015). The Challenge of Treating Superbugs. Semin. Respir. Crit. Care Med..

[10] Magiorakos A.P., Srinivasan A., Carey R.B., Carmeli Y., Falagas M.E., Giske C.G., Harbarth S., Hindler J.F., Kahlmeter G., Olsson-Liljequist B. (2012). Multidrug-resistant, extensively drug-resistant and pandrug-resistant bacteria: An international expert proposal for interim standard definitions for acquired resistance. Clin. Microbiol. Infect..

[11] Angele M.K., Faist E. (2002). Clinical review: Immunodepression in the surgical patient and increased susceptibility to infection. Crit. Care.

[12] De Waele J.J., Boelens J., Leroux-Roels I. (2020). Multidrug-resistant bacteria in ICU: Fact or myth. Curr. Opin. Anaesthesiol..

[13] Bassetti M., Righi E., Vena A., Graziano E., Russo A., Peghin M. (2018). Risk stratification and treatment of ICU-acquired pneumonia caused by multidrug- resistant/extensively drug-resistant/pandrug-resistant bacteria. Curr. Opin. Crit. Care.

[14] Blot S., Antonelli M., Arvaniti K., Blot K., Creagh-Brown B., de Lange D., De Waele J., Deschepper M., Dikmen Y., The Abdominal Sepsis Study (AbSeS) Group on behalf of the Trials Group of the European Society of Intensive Care Medicine (2019). Epidemiology of intra-abdominal infection and sepsis in critically ill patients: “AbSeS”, a multinational observational cohort study and ESICM Trials Group Project. Intensive Care Med..

[15] Bassetti S., Tschudin-Sutter S., Egli A., Osthoff M. (2022). Optimizing antibiotic therapies to reduce the risk of bacterial resistance. Eur. J. Intern. Med..

[16] Wunderink R.G., Srinivasan A., Barie P.S., Chastre J., Dela Cruz C.S., Douglas I.S., Ecklund M., Evans S.E., Evans S.R., Gerlach A.T. (2020). Antibiotic Stewardship in the Intensive Care Unit. An Official American Thoracic Society Workshop Report in Collaboration with the AACN, CHEST, CDC, and SCCM. Ann. Am. Thorac. Soc..

[17] De Waele J.J., Dhaese S. (2019). Antibiotic stewardship in sepsis management: Toward a balanced use of antibiotics for the severely ill patient. Expert Rev. Anti Infect. Ther..

[18] Shrestha J., Zahra F., Cannady J. (2023). Antimicrobial Stewardship. StatPearls.

[19] Srinivasan A. (2017). Antibiotic stewardship: Why we must, how we can. Cleve Clin. J. Med..

[20] Damiani E., Donati A., Serafini G., Rinaldi L., Adrario E., Pelaia P., Busani S., Girardis M. (2015). Effect of performance improvement programs on compliance with sepsis bundles and mortality: A systematic review and meta-analysis of observational studies. PLoS ONE.

[21] Schinkel M., Nanayakkara P.W.B., Wiersinga W.J. (2022). Sepsis Performance Improvement Programs: From Evidence Toward Clinical Implementation. Crit. Care.

[22] Evans L., Rhodes A., Alhazzani W., Antonelli M., Coopersmith C.M., French C., Machado F.R., Mcintyre L., Ostermann N., Prescott H.C. (2021). Surviving sepsis campaign: International guidelines for management of sepsis and septic shock 2021. Intensive Care Med..

[23] Méndez R., Figuerola A., Chicot M., Barrios A., Pascual N., Ramasco F., Rodríguez D., García I., von Wernitz A., Zurita N. (2022). Sepsis Code: Dodging mortality in a tertiary hospital. Rev. Esp. Quimioter..

[24] Mensa J., Barberán J., Ferrer R., Borges M., Rascado P., Maseda E., Oliver A., Marco F., Adalia R., Aguilar G. (2021). Recommendations for antibiotic selection for severe nosocomial infections. Rev. Esp. Quimioter..

[25] Peri A.M., Stewart A., Hume A., Irwin A., Harris P.N.A. (2021). New Microbiological Techniques for the Diagnosis of Bacterial Infections and Sepsis in ICU Including Point of Care. Curr. Infect. Dis. Rep..

[26] Tamma P.D., Miller M.A., Cosgrove S.E. (2019). Rethinking How Antibiotics Are Prescribed: Incorporating the 4 Moments of Antibiotic Decision Making Into Clinical Practice. JAMA.

[27] Timsit J.F., Bassetti M., Cremer O., Daikos G., de Waele J., Kallil A., Kipnis E., Kollef M., Laupland K., Paiva J.-A. (2019). Rationalizing antimicrobial therapy in the ICU: A narrative review. Intensive Care Med..

[28] Princess I., Vadala R. (2021). Clinical Microbiology in the Intensive Care Unit: Time for Intensivists to Rejuvenate this Lost Art. Indian J. Crit. Care Med..

[29] Gupta E., Saxena J., Kumar S., Sharma U., Rastogi S., Srivastava V.K., Kaushik S., Jyoti A. (2023). Fast Track Diagnostic Tools for Clinical Management of Sepsis: Paradigm Shift from Conventional to Advanced Methods. Diagnostics.

[30] Gerace E., Mancuso G., Midiri A., Poidomani S., Zummo S., Biondo C. (2022). Recent Advances in the Use of Molecular Methods for the Diagnosis of Bacterial Infections. Pathogens.

[31] Hansen G.T. (2020). Point-of-Care Testing in Microbiology: A Mechanism for Improving Patient Outcomes. Clin. Chem..

[32] Kowalska-Krochmal B., Dudek-Wicher R. (2021). The Minimum Inhibitory Concentration of Antibiotics: Methods, Interpretation, Clinical Relevance. Pathogens.

[33] Nabal Díaz S.G., Algara Robles O., García-Lechuz Moya J.M. (2022). New definitions of susceptibility categories EUCAST 2019: Clinic application. Rev. Esp. Quim..

[34] Drlica K., Zhao X. (2007). Mutant Selection Window Hypothesis Updated. Clin. Infect. Dis..

[35] Zhao X., Drlica K. (2002). Restricting the selection of antibiotic-resistant mutant bacteria: Measurement and potential use of the mutant selection window. J. Infect. Dis..

[36] Mensa J., Barberán J., Soriano A., Llinares P., Marco F., Cantón R., Bou G., González Del Castillo J., Maseda E., Azanza J.R. (2018). Antibiotic selection in the treatment of acute invasive infections by Pseudomonas aeruginosa: Guidelines by the Spanish Society of Chemotherapy. Rev. Esp. Quimioter..

[37] Schlebusch S., Graham R.M.A., Jennison A.V., Lassig-Smith M.M., Harris P.N.A., Lipman J., Ó Cuív P., Paterson D.L. (2022). Standard rectal swabs as a surrogate sample for gut microbiome monitoring in intensive care. BMC Microbiol..

[38] Montero J.G., Lerma F.Á., Galleymore P.R., Martínez M.P., Rocha L.Á., Gaite F.B., Rodríguez J.A., González M.C., Moreno I.F., Baño J.R. (2015). Combatting resistance in intensive care: The multimodal approach of the Spanish ICU «Zero Resistance» program. Crit. Care.

[39] Roberts J.A., Taccone F.S., Lipman J. (2016). Understanding PK/PD. Intensive Care Med..

[40] Roberts J.A., Paul S.K., Akova M., Bassetti M., De Waele J.J., Dimopoulos G., Kaukonen K.-M., Koulenti D., Martin C., Montravers P. (2014). DALI: Defining antibiotic levels in intensive care unit patients: Are current β-lactam antibiotic doses sufficient for critically ill patients?. Clin. Infect. Dis..

[41] Guilhaumou R., Benaboud S., Bennis Y., Dahyot-Fizelier C., Dailly E., Gandia P., Goutelle S., Lefeuvre S., Mongardon N., Roger C. (2019). Optimization of the treatment with beta-lactam antibiotics in critically ill patients—Guidelines from the French Society of Pharmacology and Therapeutics (Société Française de Pharmacologie et Thérapeutique—SFPT) and the French Society of Anaesthesia and Intensive Care Medicine (Société Française d’Anesthésie et Réanimation—SFAR). Crit. Care.

[42] Hong L.T., Downes K.J., FakhriRavari A., Abdul-Mutakabbir J.C., Kuti J.L., Jorgensen S., Young D.C., Alshaer M.H., Bassetti M., Bonomo R.A. (2023). International consensus recommendations for the use of prolonged-infusion beta-lactam antibiotics: Endorsed by the American College of Clinical Pharmacy, British Society for Antimicrobial Chemotherapy, Cystic Fibrosis Foundation, European Society of Clinical Microbiology and Infectious Diseases, Infectious Diseases Society of America, Society of Critical Care Medicine, and Society of Infectious Diseases Pharmacists: An executive summary. Pharmacotherapy.

[43] Tang R., Luo R., Wu B., Wang F., Song H., Chen X. (2021). Effectiveness and safety of adjunctive inhaled antibiotics for ventilator-associated pneumonia: A systematic review and meta-analysis of randomized controlled trials. J. Crit. Care.

[44] Kalil A.C., Metersky M.L., Klompas M., Muscedere J., Sweeney D.A., Palmer L.B., Napolitano L.M., O’Grady N.P., Bartlett J.G., Carratalà J. (2016). Management of Adults with Hospital-acquired and Ventilator-associated Pneumonia: 2016 Clinical Practice Guidelines by the Infectious Diseases Society of America and the American Thoracic Society. Clin. Infect. Dis..

[45] Tablan O.C., Anderson L.J., Besser R., Bridges C., Hajjeh R., CDC, Healthcare Infection Control Practices Advisory Committee (2004). Guidelines for preventing health-care-associated pneumonia, 2003: Recommendations of CDC and the Healthcare Infection Control Practices Advisory Committee. Morb. Mortal. Wkly. Rep. Recomm. Rep..

[46] Rello J., Solé-Lleonart C., Rouby J.J., Chastre J., Blot S., Poulakou G., Luyt C.-E., Riera J., Palmer L.B., Pereira J.M. (2017). Use of nebulized antimicrobials for the treatment of respiratory infections in invasively mechanically ventilated adults: A position paper from the European Society of Clinical Microbiology and Infectious Diseases. Clin. Microbiol. Infect..

[47] Gorham J., Taccone F.S., Hites M. (2023). How to Use Nebulized Antibiotics in Severe Respiratory Infections. Antibiotics.

[48] Abdul-Aziz M.H., Alffenaar J.W.C., Bassetti M., Bracht H., Dimopoulos G., Marriott D., Neely M.N., Paiva J.-A., Pea F., Sjovall F. (2020). Antimicrobial therapeutic drug monitoring in critically ill adult patients: A Position Paper. Intensive Care Med..

[49] Shi A.X., Qu Q., Zhuang H.H., Teng X.Q., Xu W.X., Liu Y.P., Xiao Y.W., Qu J. (2023). Individualized antibiotic dosage regimens for patients with augmented renal clearance. Front. Pharmacol..

[50] Shenoy E.S., Macy E., Rowe T., Blumenthal K.G. (2019). Evaluation and Management of Penicillin Allergy: A Review. JAMA.

[51] Stone C.A., Trubiano J., Coleman D.T., Rukasin C.R.F., Phillips E.J. (2020). The challenge of de-labeling penicillin allergy. Allergy.

[52] Caruso C., Valluzzi R.L., Colantuono S., Gaeta F., Romano A. (2021). β-Lactam Allergy and Cross-Reactivity: A Clinician’s Guide to Selecting an Alternative Antibiotic. J. Asthma Allergy.

[53] Alvarez-Cuesta E., Madrigal-Burgaleta R., Broyles A.D., Cuesta-Herranz J., Guzman-Melendez M.A., Maciag M.C., Phillips E.J., Trubiano J.A., Wong  J.T., Ansotegui I. (2022). Standards for practical intravenous rapid drug desensitization & delabeling: A WAO committee statement. World Allergy Organ. J..

[54] Savic L., Ardern-Jones M., Avery A., Cook T., Denman S., Farooque S., Garcez T., Gold R., Jay N., Krishna M.T. (2022). BSACI guideline for the set-up of penicillin allergy de-labelling services by non-allergists working in a hospital setting. Clin. Exp. Allergy.

[55] Trubiano J.A., Vogrin S., Chua K.Y.L., Bourke J., Yun J., Douglas A., Stone A.C., Yu R., Lauren G., Holmes N.E. (2020). Development and Validation of a Penicillin Allergy Clinical Decision Rule. JAMA Intern. Med..

[56] Courtemanche J., Baril L., Clément A., Bédard M.A., Plourde M., Émond M. (2022). Is it possible to identify patients at low risk of having a true penicillin allergy?. Can. J. Emerg. Med..

[57] Trubiano J.A. (2022). A Risk-Based Approach to Penicillin Allergy. Immunol. Allergy Clin. North Am..

[58] Holmes M.D., Vo N., Rafeq R., Byrne D., King M. (2023). Administration of β-lactam antibiotics to patients with reported penicillin allergy in the emergency department. Am. J. Emerg. Med..

[59] Pulcini C., Bush K., Craig W.A., Frimodt-Møller N., Grayson M.L., Mouton J.W., Turnidge J., Harbarth S., Gyssens I.C., ESCMID Study Group for Antibiotic Policies (2012). Forgotten antibiotics: An inventory in Europe, the United States, Canada, and Australia. Clin. Infect. Dis..

[60] El-Sayed Ahmed M.A.E.G., Zhong L.L., Shen C., Yang Y., Doi Y., Tian G.B. (2020). Colistin and its role in the Era of antibiotic resistance: An extended review (2000–2019). Emerg. Microbes Infect..

[61] Múñez Rubio E., Ramos Martínez A., Fernández Cruz A. (2019). Fosfomycin in antimicrobial stewardship programs. Rev. Esp. Quimioter..

[62] Rychlíčková J., Kubíčková V., Suk P., Urbánek K. (2023). Challenges of Colistin Use in ICU and Therapeutic Drug Monitoring: A Literature Review. Antibiotics.

[63] Brown G.R. (2014). Cotrimoxazole—Optimal dosing in the critically ill. Ann. Intensive Care.

[64] Tamma P.D., Aitken S.L., Bonomo R.A., Mathers A.J., van Duin D., Clancy C.J. (2023). Infectious Diseases Society of America 2023 Guidance on the Treatment of Antimicrobial Resistant Gram-Negative Infections. Clin. Infect. Dis..

[65] Yaghoubi S., Zekiy A.O., Krutova M., Gholami M., Kouhsari E., Sholeh M., Ghafouri Z., Maleki F. (2022). Tigecycline antibacterial activity, clinical effectiveness, and mechanisms and epidemiology of resistance: Narrative review. Eur. J. Clin. Microbiol. Infect. Dis..

[66] De Pascale G., Lisi L., Ciotti G.M.P., Vallecoccia M.S., Cutuli S.L., Cascarano L., Gelormini C., Bello G., Montini L., Carelli S. (2020). Pharmacokinetics of high-dose tigecycline in critically ill patients with severe infections. Ann. Intensive Care.

[67] Spellberg B., Blaser M., Guidos R.J., Boucher H.W., Bradley J.S., Infectious Diseases Society of America (IDSA) (2011). Combating antimicrobial resistance: Policy recommendations to save lives. Clin. Infect. Dis..

[68] Brown N.M., Goodman A.L., Horner C., Jenkins A., Brown E.M. (2021). Treatment of methicillin-resistant *Staphylococcus aureus* (MRSA): Updated guidelines from the UK. JAC Antimicrob. Resist..

[69] Habib G., Lancellotti P., Antunes M.J., Bongiorni M.G., Casalta J.P., Del Zotti F., Dulgheru R., El Khoury G., Erba P.A., Iung B. (2015). 2015 ESC Guidelines for the management of infective endocarditis: The Task Force for the Management of Infective Endocarditis of the European Society of Cardiology (ESC). Endorsed by: European Association for Cardio-Thoracic Surgery (EACTS), the European Association of Nuclear Medicine (EANM). Eur. Heart J..

[70] Infectious Diseases Society of America (2010). The 10 x ’20 Initiative: Pursuing a global commitment to develop 10 new antibacterial drugs by 2020. Clin. Infect. Dis..

[71] Hetzler L., Kollef M.H., Yuenger V., Micek S.T., Betthauser K.D. (2022). New antimicrobial treatment options for severe Gram-negative infections. Curr. Opin. Crit. Care.

[72] Clancy C.J., Nguyen M.H. (2022). Management of Highly Resistant Gram-Negative Infections in the Intensive Care Unit in the Era of Novel Antibiotics. Infect. Dis. Clin. N. Am..

[73] Shirley M. (2018). Ceftazidime-Avibactam: A Review in the Treatment of Serious Gram-Negative Bacterial Infections. Drugs.

[74] Matesanz M., Mensa J. (2021). Ceftazidime-Avibactam. Rev. Esp. Quimioter..

[75] Pintado V., Ruiz-Garbajosa P., Aguilera-Alonso D., Baquero-Artigao F., Bou G., Cantón R., Grau S., Gutiérrez-Gutiérrez B., Larrosa N., Machuca I. (2023). Executive summary of the consensus document of the Spanish Society of Infectious Diseases and Clinical Microbiology (SEIMC) on the diagnosis and antimicrobial treatment of infections due to carbapenem-resistant Gram-negative bacteria. Enferm. Infecc. Microbiol. Clin..

[76] Paul M., Carrara E., Retamar P., Tängdén T., Bitterman R., Bonomo R.A., de Waele J., Daikos G.L., Akova M., Harbarth S. (2022). European Society of Clinical Microbiology and Infectious Diseases (ESCMID) guidelines for the treatment of infections caused by multidrug-resistant Gram-negative bacilli (endorsed by European society of intensive care medicine). Clin. Microbiol. Infect..

[77] Bassetti M., Garau J. (2021). Current and future perspectives in the treatment of multidrug-resistant Gram-negative infections. J. Antimicrob. Chemother..

[78] Soriano A., Carmeli Y., Omrani A.S., Moore L.S.P., Tawadrous M., Irani P. (2021). Ceftazidime-Avibactam for the Treatment of Serious Gram-Negative Infections with Limited Treatment Options: A Systematic Literature Review. Infect. Dis. Ther..

[79] Soriano A., Montravers P., Bassetti M., Klyasova G., Daikos G., Irani P., Stone G., Chambers R., Peeters P., Shah M. (2023). The Use and Effectiveness of Ceftazidime–Avibactam in Real-World Clinical Practice: EZTEAM Study. Infect. Dis. Ther..

[80] Swaminathan S., Routray A., Mane A. (2022). Early and Appropriate Use of Ceftazidime-Avibactam in the Management of Multidrug-Resistant Gram-Negative Bacterial Infections in the Indian Scenario. Cureus.

[81] Jorgensen S.C.J., Trinh T.D., Zasowski E.J., Lagnf A.M., Bhatia S., Melvin S.M., E Steed M., Simon S.P., Estrada S.J., Morrisette T. (2019). Real-World Experience with Ceftazidime-Avibactam for Multidrug-Resistant Gram-Negative Bacterial Infections. Open Forum Infect. Dis..

[82] Castón J.J., Cano A., Pérez-Camacho I., Aguado J.M., Carratalá J., Ramasco F., Soriano A., Pintado V., Castelo-Corral L., Sousa A. (2022). Impact of ceftazidime/avibactam versus best available therapy on mortality from infections caused by carbapenemase-producing Enterobacterales (CAVICOR study). J. Antimicrob. Chemother..

[83] Mikhail S., Singh N.B., Kebriaei R., Rice S.A., Stamper K.C., Castanheira M., Rybak M.J. (2019). Evaluation of the Synergy of Ceftazidime-Avibactam in Combination with Meropenem, Amikacin, Aztreonam, Colistin, or Fosfomycin against Well-Characterized Multidrug-Resistant *Klebsiella pneumoniae* and *Pseudomonas aeruginosa*. Antimicrob. Agents Chemother..

[84] Wang L.T., Lin W.T., Lai C.C., Wang Y.H., Chen C.H., Chang Y.T., Chen C.-H., Wang C.-Y. (2021). The safety of ceftolozane-tazobactam for the treatment of acute bacterial infections: A systemic review and meta-analysis. Ther. Adv. Drug Saf..

[85] Candel F.J., del Castillo J.G., Jiménez A.J., Matesanz M. (2022). Ceftolozane-tazobactam in nosocomial pneumonia. Rev. Esp. Quimioter..

[86] Timsit J.F., Huntington J.A., Wunderink R.G., Shime N., Kollef M.H., Kivistik Ü., Nováček M., Réa-Neto A., Martin-Loeches 9 I., Yu B. (2021). Ceftolozane/tazobactam versus meropenem in patients with ventilated hospital-acquired bacterial pneumonia: Subset analysis of the ASPECT-NP randomized, controlled phase 3 trial. Crit. Care.

[87] Puzniak L., Dillon R., Palmer T., Collings H., Enstone A. (2021). Real-world use of ceftolozane/tazobactam: A systematic literature review. Antimicrob. Resist. Infect. Control.

[88] Sansone P., Giaccari L.G., Coppolino F., Aurilio C., Barbarisi A., Passavanti M.B., Pota V., Pace M.C. (2022). Cefiderocol for Carbapenem-Resistant Bacteria: Handle with Care! A Review of the Real-World Evidence. Antibiotics.

[89] Maseda E., Suárez de la Rica A. (2022). The role of cefiderocol in clinical practice. Rev. Esp. Quimioter..

[90] Bassetti M., Echols R., Matsunaga Y., Ariyasu M., Doi Y., Ferrer R., Lodise T.P., Naas T., Niki Y., Paterson D.L. (2021). Efficacy and safety of cefiderocol or best available therapy for the treatment of serious infections caused by carbapenem-resistant Gram-negative bacteria (CREDIBLE-CR): A randomised, open-label, multicentre, pathogen-focused, descriptive, phase 3 trial. Lancet Infect. Dis..

[91] Viale P., Sandrock C.E., Ramirez P., Rossolini G.M., Lodise T.P. (2023). Treatment of critically ill patients with cefiderocol for infections caused by multidrug-resistant pathogens: Review of the evidence. Ann. Intensive Care.

[92] Corcione S., Lupia T., Maraolo A.E., Mornese Pinna S., Gentile I., De Rosa F.G. (2019). Carbapenem-sparing strategy: Carbapenemase, treatment, and stewardship. Curr. Opin. Infect. Dis..

[93] Jean S.S., Harnod D., Hsueh P.R. (2022). Global Threat of Carbapenem-Resistant Gram-Negative Bacteria. Front. Cell Infect. Microbiol..

[94] Arulkumaran N., Routledge M., Schlebusch S., Lipman J., Conway Morris A. (2020). Antimicrobial-associated harm in critical care: A narrative review. Intensive Care Med..

[95] Harris P.N.A., Tambyah P.A., Lye D.C., Mo Y., Lee T.H., Yilmaz M., Alenazi T.H., Arabi Y., Falcone M., Bassetti M. (2018). Effect of Piperacillin-Tazobactam vs Meropenem on 30-Day Mortality for Patients with *E. coli* or *Klebsiella pneumoniae* Bloodstream Infection and Ceftriaxone Resistance: A Randomized Clinical Trial. JAMA.

[96] Montravers P., Bassetti M. (2018). The ideal patient profile for new beta-lactam/beta-lactamase inhibitors. Curr. Opin. Infect. Dis..

[97] Kollef M.H., Shorr A.F., Bassetti M., Timsit J.F., Micek S.T., Michelson A.P., Garnacho-Montero J. (2021). Timing of antibiotic therapy in the ICU. Crit. Care.

[98] De Waele J.J., Schouten J., Beovic B., Tabah A., Leone M. (2020). Antimicrobial de-escalation as part of antimicrobial stewardship in intensive care: No simple answers to simple questions—A viewpoint of experts. Intensive Care Med..

[99] Strich J.R., Heil E.L., Masur H. (2020). Considerations for Empiric Antimicrobial Therapy in Sepsis and Septic Shock in an Era of Antimicrobial Resistance. J. Infect. Dis..

[100] Dequin P.F., Aubron C., Faure H., Garot D., Guillot M., Hamzaoui O., Lemiale V., Maizel J., Mootien J.Y., Osman D. (2023). The place of new antibiotics for Gram-negative bacterial infections in intensive care: Report of a consensus conference. Ann. Intensive Care.

[101] Miller W.D., Keskey R., Alverdy J.C. (2020). Sepsis and the Microbiome: A Vicious Cycle. J. Infect. Dis..

[102] Prechter F., Katzer K., Bauer M., Stallmach A. (2017). Sleeping with the enemy: Clostridium difficile infection in the intensive care unit. Crit. Care.

[103] Morel C.M., de Kraker M.E.A., Harbarth S., The Enhanced Surveillance Expert Consensus Group (CANSORT-SCI) (2021). Surveillance of Resistance to New Antibiotics in an Era of Limited Treatment Options. Front. Med..

[104] Niederman M.S., Baron R.M., Bouadma L., Calandra T., Daneman N., DeWaele J., Kollef M.H., Lipman J., Nair G.B. (2021). Initial antimicrobial management of sepsis. Crit. Care.

[105] Pea F., Viale P. (2006). The antimicrobial therapy puzzle: Could pharmacokinetic-pharmacodynamic relationships be helpful in addressing the issue of appropriate pneumonia treatment in critically ill patients?. Clin. Infect. Dis..

[106] Kumar A. (2014). An alternate pathophysiologic paradigm of sepsis and septic shock: Implications for optimizing antimicrobial therapy. Virulence.

[107] Danjean M., Hobson C.A., Gits-Muselli M., Courroux C., Monjault A., Bonacorsi S., Birgy A. (2022). Evaluation of the inoculum effect of new antibiotics against carbapenem-resistant enterobacterales. Clin. Microbiol. Infect..

[108] Heffernan A.J., Mohd Sazlly Lim S., Lipman J., Roberts J.A. (2021). A personalised approach to antibiotic pharmacokinetics and pharmacodynamics in critically ill patients. Anaesth. Crit. Care Pain. Med..

[109] Zaragoza R., Vidal-Cortés P., Aguilar G., Borges M., Diaz E., Ferrer R., Maseda E., Nieto M., Nuvials F.X., Ramirez P. (2020). Update of the treatment of nosocomial pneumonia in the ICU. Crit. Care.

[110] Di Franco S., Alfieri A., Fiore M., Fittipaldi C., Pota V., Coppolino F., Sansone P., Pace M.C., Passavanti M.B. (2022). A Literature Overview of Secondary Peritonitis Due to Carbapenem-Resistant Enterobacterales (CRE) in Intensive Care Unit (ICU) Patients. Antibiotics.

[111] Heidary M., Khosravi A.D., Khoshnood S., Nasiri M.J., Soleimani S., Goudarzi M. (2018). Daptomycin. J. Antimicrob. Chemother..

[112] Thomas-Rüddel D.O., Schlattmann P., Pletz M., Kurzai O., Bloos F. (2022). Risk Factors for Invasive Candida Infection in Critically Ill Patients: A Systematic Review and Meta-analysis. Chest.

[113] Keane S., Geoghegan P., Povoa P., Nseir S., Rodriguez A., Martin-Loeches I. (2018). Systematic review on the first line treatment of amphotericin B in critically ill adults with candidemia or invasive candidiasis. Expert Rev. Anti Infect. Ther..

[114] Burillo A., Bouza E. (2022). Faster infection diagnostics for intensive care unit (ICU) patients. Expert Rev. Mol. Diagn..

[115] Méndez Hernández R., Ramasco Rueda F. (2023). Biomarkers as Prognostic Predictors and Therapeutic Guide in Critically Ill Patients: Clinical Evidence. J. Pers. Med..

[116] Vincent J.L., Bassetti M., François B., Karam G., Chastre J., Torres A., Roberts J.A., Taccone F.S., Rello J., Calandra T. (2016). Advances in antibiotic therapy in the critically ill. Crit. Care.

[117] Ulldemolins M., Nuvials X., Palomar M., Masclans J.R., Rello J. (2011). Appropriateness is critical. Crit. Care Clin..

[118] Mahmoudi L., Niknam R., Mousavi S., Ahmadi A., Honarmand H., Ziaie S., Mojtahedzadeh M. (2013). Optimal Aminoglycoside Therapy Following the Sepsis: How Much Is Too Much?. Iran. J. Pharm. Res..

[119] Kumar A., Roberts D., Wood K.E., Light B., Parrillo J.E., Sharma S., Suppes R., Feinstein D., Zanotti S., Taiberg L. (2006). Duration of hypotension before initiation of effective antimicrobial therapy is the critical determinant of survival in human septic shock. Crit. Care Med..

[120] Ferrer R., Martin-Loeches I., Phillips G., Osborn T.M., Townsend S., Dellinger R.P., Artigas A., Schorr C., Levy M.M. (2014). Empiric antibiotic treatment reduces mortality in severe sepsis and septic shock from the first hour: Results from a guideline-based performance improvement program. Crit. Care Med..

[121] Im Y., Kang D., Ko R.E., Lee Y.J., Lim S.Y., Park S., Na S.J., Chung C.R., Park M.H., Oh D.K. (2022). Time-to-antibiotics and clinical outcomes in patients with sepsis and septic shock: A prospective nationwide multicenter cohort study. Crit. Care.

[122] Peltan I.D., Mitchell K.H., Rudd K.E., Mann B.A., Carlbom D.J., Hough C.L., Rea T.D., Brown S.M. (2017). Physician Variation in Time to Antimicrobial Treatment for Septic Patients Presenting to the Emergency Department. Crit. Care Med..

[123] Roberts R.J., Alhammad A.M., Crossley L., Anketell E., Wood L., Schumaker G., Garpestad E., Devlin J.W. (2017). A survey of critical care nurses’ practices and perceptions surrounding early intravenous antibiotic initiation during septic shock. Intensive Crit. Care Nurs..

[124] Pak T.R., Rhee C., Klompas M. (2022). Timing and Spectrum of Antibiotic Treatment for Suspected Sepsis and Septic Shock: Why so Controversial?. Infect. Dis. Clin. North Am..

[125] Rothrock S.G., Cassidy D.D., Barneck M., Schinkel M., Guetschow B., Myburgh C., Nguyen L., Earwood R., Nanayakkara P.W., Panday R.S.N. (2020). Outcome of Immediate Versus Early Antibiotics in Severe Sepsis and Septic Shock: A Systematic Review and Meta-analysis. Ann. Emerg. Med..

[126] Rhee C., Chiotos K., Cosgrove S.E., Heil E.L., Kadri S.S., Kalil A.C., Gilbert D.N., Masur H., Septimus E.J., A Sweeney D. (2021). Infectious Diseases Society of America Position Paper: Recommended Revisions to the National Severe Sepsis and Septic Shock Early Management Bundle (SEP-1) Sepsis Quality Measure. Clin. Infect. Dis..

[127] Weinberger J., Rhee C., Klompas M. (2020). A Critical Analysis of the Literature on Time-to-Antibiotics in Suspected Sepsis. J. Infect. Dis..

[128] Montravers P., Blot S., Dimopoulos G., Eckmann C., Eggimann P., Guirao X., Paiva J.A., Sganga G., De Waele J. (2016). Therapeutic management of peritonitis: A comprehensive guide for intensivists. Intensive Care Med..

[129] Ordoñez C.A., Caicedo Y., Parra M.W., Rodríguez-Holguín F., Serna J.J., Salcedo A., Franco M.J., Toro L.E., Pino L.F., Guzmán-Rodríguez M. (2021). Evolution of damage control surgery in non-traumatic abdominal pathology: A light in the darkness. Colomb. Med..

[130] De Waele J.J., Girardis M., Martin-Loeches I. (2022). Source control in the management of sepsis and septic shock. Intensive Care Med..

[131] De Pascale G., Antonelli M., Deschepper M., Arvaniti K., Blot K., Brown B.C., de Lange D., De Waele J., Dikmen Y., Dimopoulos G. (2022). Poor timing and failure of source control are risk factors for mortality in critically ill patients with secondary peritonitis. Intensive Care Med..

[132] Obst W., Esser T., Kaasch A.J., Geginat G., Meyer F., Croner R.S., Keitel V. (2022). The Need of Antimicrobial Stewardship in Post-Operative Infectious Complications of Abdominal Surgery. Visc. Med..

[133] Maseda E., Suarez-de-la-Rica A., Anillo V., Tamayo E., García-Bernedo C.A., Ramasco F., Villagran M.-J., Maggi G., Gimenez M.-J., Aguilar L. (2015). Procalcitonin-guided therapy may reduce length of antibiotic treatment in intensive care unit patients with secondary peritonitis: A multicenter retrospective study. J. Crit. Care.

[134] Montravers P., Tubach F., Lescot T., Veber B., Esposito-Farèse M., Seguin P., Paugam C., Lepape A., Meistelman C., For the DURAPOP Trial Group (2018). Short-course antibiotic therapy for critically ill patients treated for postoperative intra-abdominal infection: The DURAPOP randomised clinical trial. Intensive Care Med..

[135] Bass G.A., Dzierba A.L., Taylor B., Lane-Fall M., Kaplan L.J. (2022). Tertiary peritonitis: Considerations for complex team-based care. Eur. J. Trauma. Emerg. Surg..

[136] Marques H.S., Araújo G.R.L., da Silva F.A.F., de Brito B.B., Versiani P.V.D., Caires J.S., Milet T.d.C., de Melo F.F. (2021). Tertiary peritonitis: A disease that should not be ignored. World J. Clin. Cases.

[137] Prest J., Nguyen T., Rajah T., Prest A.B., Sathananthan M., Jeganathan N. (2022). Sepsis-Related Mortality Rates and Trends Based on Site of Infection. Crit. Care Explor..

[138] Cavaillon J., Singer M., Skirecki T. (2020). Sepsis therapies: Learning from 30 years of failure of translational research to propose new leads. EMBO Mol. Med..

[139] Baggs J., Jernigan J.A., Halpin A.L., Epstein L., Hatfield K.M., McDonald L.C. (2018). Risk of Subsequent Sepsis within 90 Days after a Hospital Stay by Type of Antibiotic Exposure. Clin. Infect. Dis..

[140] Teshome B.F., Vouri S.M., Hampton N., Kollef M.H., Micek S.T. (2019). Duration of Exposure to Antipseudomonal β-Lactam Antibiotics in the Critically Ill and Development of New Resistance. Pharmacotherapy.

[141] Raman K., Nailor M.D., Nicolau D.P., Aslanzadeh J., Nadeau M., Kuti J.L. (2013). Early Antibiotic Discontinuation in Patients with Clinically Suspected Ventilator-Associated Pneumonia and Negative Quantitative Bronchoscopy Cultures. Crit. Care Med..

[142] Sawyer R.G., Claridge J.A., Nathens A.B., Rotstein O.D., Duane T.M., Evans H.L., Cook C.H., O’neill P.J., Mazuski J.E., Askari R. (2015). Trial of Short-Course Antimicrobial Therapy for Intraabdominal Infection. N. Engl. J. Med..

[143] Yahav D., Franceschini E., Koppel F., Turjeman A., Babich T., Bitterman R., Neuberger A., Ghanem-Zoubi N., Santoro A., Eliakim-Raz N. (2019). Seven Versus 14 Days of Antibiotic Therapy for Uncomplicated Gram-negative Bacteremia: A Noninferiority Randomized Controlled Trial. Clin. Infect. Dis..

[144] Thorlacius-Ussing L., Andersen C.Ø., Frimodt-Møller N., Knudsen I.J.D., Lundgren J., Benfield T.L. (2019). Efficacy of seven and fourteen days of antibiotic treatment in uncomplicated *Staphylococcus aureus* bacteremia (SAB7): Study protocol for a randomized controlled trial. Trials.

[145] Kim C.J., Song K.H., Park K.H., Kim M., Choe P.G., Oh M d et a.l. (2019). Impact of antimicrobial treatment duration on outcome of Staphylococcus aureus bacteraemia: A cohort study. Clin. Microbiol. Infect..

[146] Chastre J., Wolff M., Fagon J.Y., Chevret S., Thomas F., Wermert D., Clementi E., Gonzalez J., Jusserand D., Asfar D. (2003). Comparison of 8 vs 15 Days of Antibiotic Therapy for Ventilator-Associated Pneumonia in Adults: A Randomized Trial. JAMA.

[147] Bouglé A., Tuffet S., Federici L., Leone M., Monsel A., Dessalle T., Amour J., Dahyot-Fizelier C., Barbier F., Luyt C.-E. (2022). Comparison of 8 versus 15 days of antibiotic therapy for *Pseudomonas aeruginosa* ventilator-associated pneumonia in adults: A randomized, controlled, open-label trial. Intensive Care Med..

[148] Metersky M.L., Klompas M., Kalil A.C. (2023). Less Is More: A 7-Day Course of Antibiotics Is the Evidence-Based Treatment for *Pseudomonas aeruginosa* Ventilator-Associated Pneumonia. Clin. Infect. Dis..

[149] Rouzé A., Martin-Loeches I., Povoa P., Makris D., Artigas A., Bouchereau M., Lambiotte F., Metzelard M., Cuchet P., Geronimi C.B. (2021). Relationship between SARS-CoV-2 infection and the incidence of ventilator-associated lower respiratory tract infections: A European multicenter cohort study. Intensive Care Med..

[150] Assicot M., Bohuon C., Gendrel D., Raymond J., Carsin H., Guilbaud J. (1993). High serum procalcitonin concentrations in patients with sepsis and infection. Lancet.

[151] Rau B., Krüger C.M., Schilling M.K. (2004). Procalcitonin: Improved biochemical severity stratification and postoperative monitoring in severe abdominal inflammation and sepsis. Langenbeck’s Arch. Surg..

[152] Bouadma L., Luyt C.E., Tubach F., Cracco C., Alvarez A., Schwebel C., Schortgen F., Lasocki S., Veber B., Dehoux M. (2010). Use of procalcitonin to reduce patients’ exposure to antibiotics in intensive care units (PRORATA trial): A multicentre randomised controlled trial. Lancet.

[153] De Jong E., Van Oers J.A., Beishuizen A., Vos P., Vermeijden W.J., Haas L.E., Loef B.G., Dormans T., van Melsen G.C., Kluiters Y.C. (2016). Efficacy and safety of procalcitonin guidance in reducing the duration of antibiotic treatment in critically ill patients: A randomised, controlled, open-label trial. Lancet Infect. Dis..

[154] Larsson A., Tydén J., Johansson J., Lipcsey M., Bergquist M., Kultima K., Mandic-Havelka A. (2020). Calprotectin is superior to procalcitonin as a sepsis marker and predictor of 30-day mortality in intensive care patients. Scand. J. Clin. Lab. Investig..

[155] Parke Å., Unge C., Yu D., Sundén-Cullberg J., Strålin K. (2023). Plasma calprotectin as an indicator of need of transfer to intensive care in patients with suspected sepsis at the emergency department. BMC Emerg. Med..

[156] Sartelli M., Chichom-Mefire A., Labricciosa F.M., Hardcastle T., Abu-Zidan F.M., Adesunkanmi A.K., Ansaloni A., Bala M., Balogh Z.J., Beltrán M.A. (2017). The management of intra-abdominal infections from a global perspective: 2017 WSES guidelines for management of intra-abdominal infections. World J. Emerg. Surg..

[157] Zilberberg M.D., Shorr A.F., Micek S.T., Vazquez-Guillamet C., Kollef M.H. (2014). Multi-drug resistance, inappropriate initial antibiotic therapy and mortality in Gram-negative severe sepsis and septic shock: A retrospective cohort study. Crit. Care.

[158] Huang C.T., Tsai Y.J., Tsai P.R., Yu C.J., Ko W.J. (2016). Severe Sepsis and Septic Shock: Timing of Septic Shock Onset Matters. Shock.

[159] Vazquez-Guillamet C., Scolari M., Zilberberg M.D., Shorr A.F., Micek S.T., Kollef M. (2014). Using the number needed to treat to assess appropriate antimicrobial therapy as a determinant of outcome in severe sepsis and septic shock. Crit. Care Med..

[160] Panidis D., Markantonis S.L., Boutzouka E., Karatzas S., Baltopoulos G. (2005). Penetration of gentamicin into the alveolar lining fluid of critically ill patients with ventilator-associated pneumonia. Chest.

[161] Tsuji B.T., Pogue J.M., Zavascki A.P., Paul M., Daikos G.L., Forrest A., Giacobbe D.R., Viscoli C., Giamarellou H., Karaiskos I. (2019). International Consensus Guidelines for the Optimal Use of the Polymyxins: Endorsed by the American College of Clinical Pharmacy (ACCP), European Society of Clinical Microbiology and Infectious Diseases (ESCMID), Infectious Diseases Society of America (IDSA), International Society for Anti-infective Pharmacology (ISAP), Society of Critical Care Medicine (SCCM), and Society of Infectious Diseases Pharmacists (SIDP). Pharmacotherapy.

[162] Bassetti M., Peghin M. (2020). How to manage KPC infections. Ther. Adv. Infect. Dis..

[163] Cano A., Gutiérrez-Gutiérrez B., Machuca I., Gracia-Ahufinger I., Pérez-Nadales E., Causse M., Castón J.J., Guzman-Puche J., Torre-Giménez J., Kindelán K. (2018). Risks of Infection and Mortality among Patients Colonized with *Klebsiella pneumoniae* Carbapenemase-Producing *K. pneumoniae*: Validation of Scores and Proposal for Management. Clin. Infect. Dis..

[164] Burillo A., Muñoz P., Bouza E. (2019). Risk stratification for multidrug-resistant Gram-negative infections in ICU patients. Curr. Opin. Infect. Dis..

[165] Giannella M., Pascale R., Gutiérrez-Gutiérrez B., Cano A., Viale P. (2019). The use of predictive scores in the management of patients with carbapenem-resistant *Klebsiella pneumoniae* infection. Expert Rev. Anti Infect. Ther..

[166] Wilson G.M., Suda K.J., Fitzpatrick M.A., Bartle B., Pfeiffer C.D., Jones M., Rubin M.A., Perencevich E., Evans M., Evans C.T. (2021). Risk Factors Associated with Carbapenemase-Producing Carbapenem-Resistant Enterobacteriaceae Positive Cultures in a Cohort of US Veterans. Clin. Infect. Dis..

[167] De Waele J.J., Coccolini F., Lagunes L., Maseda E., Rausei S., Rubio-Perez I., Theodorakopoulou M., Arvanti K. (2023). Optimized Treatment of Nosocomial Peritonitis. Antibiotics.

